# Differential Action between Schisandrin A and Schisandrin B in Eliciting an Anti-Inflammatory Action: The Depletion of Reduced Glutathione and the Induction of an Antioxidant Response

**DOI:** 10.1371/journal.pone.0155879

**Published:** 2016-05-19

**Authors:** Pou Kuan Leong, Hoi Shan Wong, Jihang Chen, Wing Man Chan, Hoi Yan Leung, Kam Ming Ko

**Affiliations:** Division of Life Science, The Hong Kong University of Science & Technology, Clear Water Bay, Hong Kong SAR, China; Yong Loo Lin School of Medicine, National University of Singapore, SINGAPORE

## Abstract

Schisandrin A (Sch A) and schisandrin B (Sch B) are active components of Schisandrae Fructus. We compared the biochemical mechanism underlying the anti-inflammatory action of Sch A and Sch B, using cultured lipopolysaccharide (LPS)-stimulated RAW264.7 macrophages and concanavalin (ConA)-stimulated mouse splenocytes. Pre-incubation with Sch A or Sch B produced an anti-inflammatory action in LPS-stimulated RAW264.7 cells, as evidenced by the inhibition of the pro-inflammatory c-Jun N-terminal kinases/p38 kinase/nuclear factor-κB signaling pathway as well as the suppression of various pro-inflammatory cytokines and effectors, with the extent of inhibition by Sch A being more pronounced. The greater activity of Sch A in anti-inflammatory response was associated with a greater decrease in cellular reduced glutathione (GSH) level and a greater increase in glutathione S-transferase activity than corresponding changes produced by Sch B. However, upon incubation, only Sch B resulted in the activation of the nuclear factor (erythroid-derived 2)-like factor 2 and the induction of a significant increase in the expression of thioredoxin (TRX) in RAW264.7 cells. The Sch B-induced increase in TRX expression was associated with the suppression of pro-inflammatory cytokines and effectors in LPS-stimulated macrophages. Studies in a mouse model of inflammation (carrageenan-induced paw edema) indicated that while long-term treatment with either Sch A or Sch B suppressed the extent of paw edema, only acute treatment with Sch A produced a significant degree of inhibition on the inflammatory response. Although only Sch A decreased the cellular GSH level and suppressed the release of pro-inflammatory cytokines and cell proliferation in ConA-simulated splenocytes *in vitro*, both Sch A and Sch B treatments, while not altering cellular GSH levels, suppressed ConA-stimulated splenocyte proliferation *ex vivo*. These results suggest that Sch A and Sch B may act differentially on activating GST/ depleting cellular GSH and inducing an antioxidant response involved in their anti-inflammatory actions.

## Introduction

The inflammatory response, as an integral part of innate immunity, enables the removal of pathogens and the clearance of injured cells by means of a complex interplay involving resident immune cells (resident macrophages) and patrolling immune cells (monocytes/macrophages, natural killer cells and neutrophils, etc) via a series of biochemical events [1]. In brief, resident macrophages are activated by the damage-associated molecular patterns (such as ATP, DNA and uric acid) from damaged cells as well as pathogen-associated molecular pattern [such as lipopolysaccharide (LPS), lipoteichoic acid and peptidoglycan] on the surface of pathogens, with a resultant release of cytokines as well as vasodilation at the injured site [2]. In response to the chemoattractants released from resident macrophages at the injured site, polymorphonuclear neutrophils, which are the most abundant circulating leukocytes, promptly infiltrate into the inflamed site and promote the extravasation of inflammatory monocytes [1, 3]. The recruited monocytes then differentiate into mature macrophages, which subsequently lead to the phagocytosis of pathogen, the promotion of wound healing and the resolution of inflammation [1, 3].

Despite the fact that the inflammatory response is beneficial to the host, a chronic and low-grade inflammation was found to be associated with the pathogenesis of a number of diseases, such as inflammatory bowel disease [4], cardiovascular diseases [5] and cancers [6]. Recent studies have suggested that the oxidative stress arising from chronic inflammatory responses may cause sustained tissue damage, which can further amplify the inflammatory response. This vicious cycle has been implicated in the pathogenesis of a number of diseases [7–8] and “inflamm-aging” [9–10]. Recent experimental evidence has suggested that the increased production of reactive oxygen species in T lymphocytes can alter cellular signaling transduction pathways, such as the modulation of T cell receptor signaling, the activation of the Src homology region 2 domain-containing phosphatase-1 (SHP-1) and the inactivation of tyrosine-specific protein phosphatase (PTPase) activities, with the resultant immunosenescence and other adverse effects [11]. In this regard, the activation of an antioxidant response, which counteracts oxidative stress, is likely to produce a beneficial effect against inflammation. The cellular antioxidant system, in which the reduced form of glutathione (GSH), glutathione-related enzymes [such as glutathione S-transferase (GST)] and thioredoxin (TRX) play a critical role in the defense against oxidant-induced injuries [12–13], is regulated by a redox sensitive transcription factor, namely nuclear factor (erythroid-derived 2)-like 2 (Nrf2) [14–15]. The search for anti-inflammatory agents, particularly those with the ability to induce a Nrfs-mediated antioxidant response, is urgently in need.

**Fig 1:**
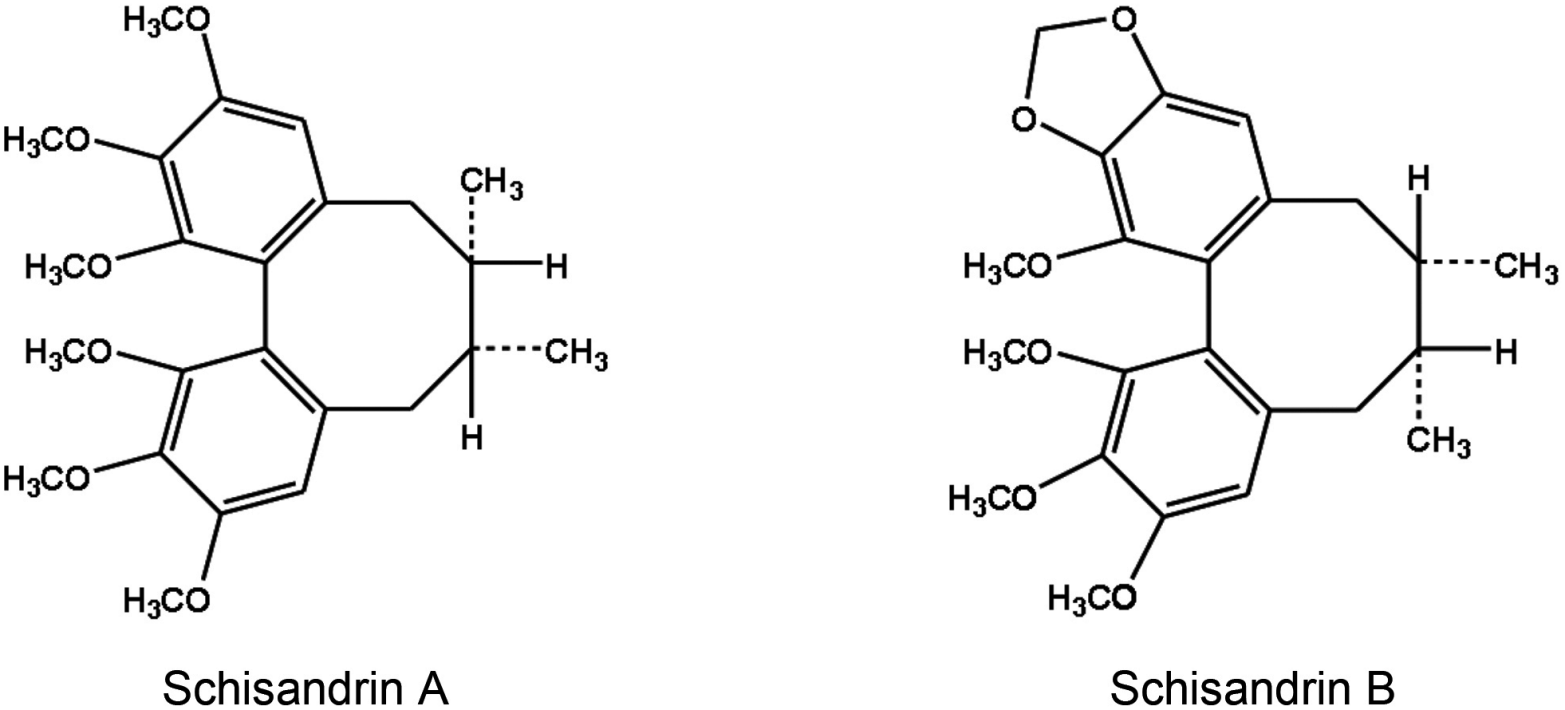
Chemical structures of Schisandrin A and Schisandrin B.

Traditional Chinese medicine, which has a long history of use in promoting and maintaining health, may offer a promising prospect for the management of inflammation. Schisandrae Fructus (FS), a traditional Chinese herb clinically prescribed for the treatment of hepatitis, contains two species, namely the dried fruits of *Schisandra sphenanthera* and *Schisandra chinensis*. Schisandrin A (Sch A) and schisandrin B (Sch B) are the most abundant active dibenzocyclooctadiene lignans in the fruits of *Schisandra sphenanthera* and *Schisandra chinensis*, respectively (Fig 1). While the biological activity of Sch A has not been fully investigated, the finding that the extent of inflammation is ameliorated by the incubation with a Sch A-enriched extract of *Schisandra sphenanthera* in ultraviolet B-irradiated HaCaT keratinocytes, is suggestive of anti-inflammatory properties of Sch A [16]. Sch B was found to activate a Nrf2-mediated glutathione antioxidant response and protect against oxidant-induced injuries in cultured cells and in various tissues of rodents [12–13]. The anti-inflammatory activity of Sch B has also been demonstrated in LPS-activated RAW264.7 macrophages [17] and concanavalin A (Con A)-activated T-lymphocytes [18]. However, the inter-relationship between the Sch B-induced glutathione antioxidant response and anti-inflammatory activity has not been investigated.

**Fig 2:**
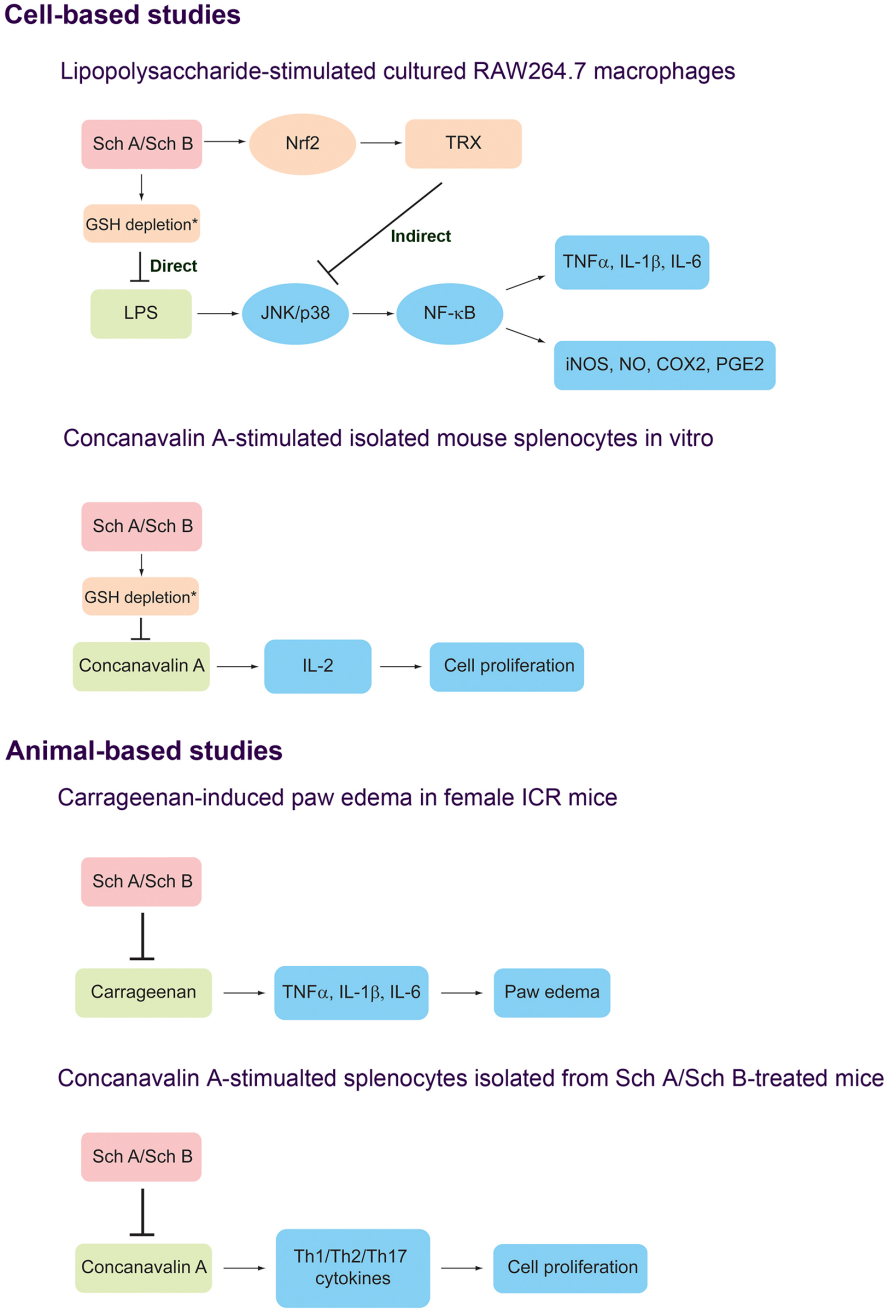
Hypotheses of the present study.

Given the differential abilities of Sch A and Sch B in triggering the redox signaling pathway [19], it remains to be determined whether or not Sch A and Sch B act through the same anti-inflammatory signaling pathway. In the present study, we hypothesized that while Sch A can produce a direct anti-inflammatory action, Sch B may act indirectly to produce its anti-inflammatory action. To test our hypothesis, we examined the effects of Sch A and Sch B on the pro-inflammatory signal transduction pathway (JNK1/2, p38 and NK-κB), on pro-inflammatory cytokines [tumor necrosis factor-α (TNF-α), interleukin 1β (IL-1β) and on interleukin 6 (IL-6)] and inflammatory effectors [inducible nitric oxide synthase (iNOS), nitric oxide, cyclooxygenase 2 (COX2) and prostaglandin E2 (PGE2)] in LPS-activated RAW264.7 macrophages (Fig 2). The effects of Sch A and Sch B on cell proliferation and on the release of cytokines in Con A-activated lymphocytes were also examined (Fig 2). To examine the role of the antioxidants in the anti-inflammatory activity, the effects of Sch A and Sch B on the antioxidant response (Nrf2 activation and TRX induction) in relation to the aforementioned pro-inflammatory parameters were investigated (Fig 2). If Sch A and Sch B act through independent pathways, it would also be interesting to investigate whether Sch A and Sch B can produce a synergistic effect in anti-inflammation. Effects of Sch A and Sch B, alone or in combination, on the aforementioned biochemical parameters were therefore examined. To confirm the results obtained from cell-based studies, the effects of Sch A and Sch B, alone or in combination, on carrageenan-induced paw edema and Con A-activated isolated spenocytes in ICR mice were also investigated (Fig 2).

## Materials and Methods

### Chemicals and reagents

Dulbecco's Modified Eagle Medium (DMEM), fetal bovine serum (FBS), mouse TNF-, IL-1β, IL-2 ELISA kit, Lipofectamine LTX & PLUS reagent, Lipofectamin RNAiMAX reagent, BLOCK-iTTM Fluorescent Oligo, PureLink RNA Mini Kit, SuperScript VILO Master Mix, customized TaqMan array plates and TaqMan universal PCR Master Mix were obtained from Life Technologies (Grand Island, NY, USA). Histopaque-1083, RPMI 1640 medium, sodium pyruvate, LPS, proteinase inhibitor cocktails and phosphatase inhibitor cocktails were purchased from Sigma-Aldrich Co (St. Louis, MO, USA). Sch A and Sch B were prepared as previously described [19]. All other chemicals were of analytical grade.

### Cell culture of RAW264.7 macrophages

A murine RAW264.7 macrophage cell line was purchased from American Type Culture Collection (Rockville, MD). RAW264.7 cells were cultured in a monolayer using DMEM supplemented with 10% FBS, 100 units/mL penicillin, 0.1 mg/mL streptomycin. RAW 264.7 and isolated peritoneal macrophages were kept at 37°C in a humidified atmosphere of air and 5% CO_2_. RAW264.7 cells used for the experiments were seeded at a density of 1.25×10^5^ cell/mL on a 12 or 96-well culture plate and were allowed to grow to 60–80% confluence within 24 h prior to incubation with the test compounds.

### Animal care

Adult Imprinting Control Region (ICR) mice (8–10 weeks old, 20–25 g) were maintained under a 12-h dark/light cycle at about 22°C, and allowed food and water *ad libitum* in the Animal and Plant Care Facility at the Hong Kong University of Science and Technology (HKUST). All experimental protocols were approved by the University Committee on Research Practice at the HKUST on 7 April 2013 (reference number: SC019).

### Isolation of splenocytes from mouse spleen

Adult female ICR mice were sacrificed by cardiac excision under ketamine chloride-induced anesthesia. Splenic tissue obtained from mice was teased with a plastic syringe in a culture dish (60 mm) containing 10 mL of RPMI-1640 medium, and it was gently strained through a 200 mesh stainless steel sieve to remove clumps to produce a cell suspension. The cell suspension was washed 3 times with RMPI-1640 medium by centrifugation and re-suspension, and the cells were finally resuspended in RPMI-1640 medium supplemented with 10% FBS at a concentration of 5 × 10^6^ viable cells/mL. The viability of isolated splenocytes was determined by Trypan Blue exclusion.

### Sch A/Sch B incubation and LPS activation in macrophages

**Fig 3:**
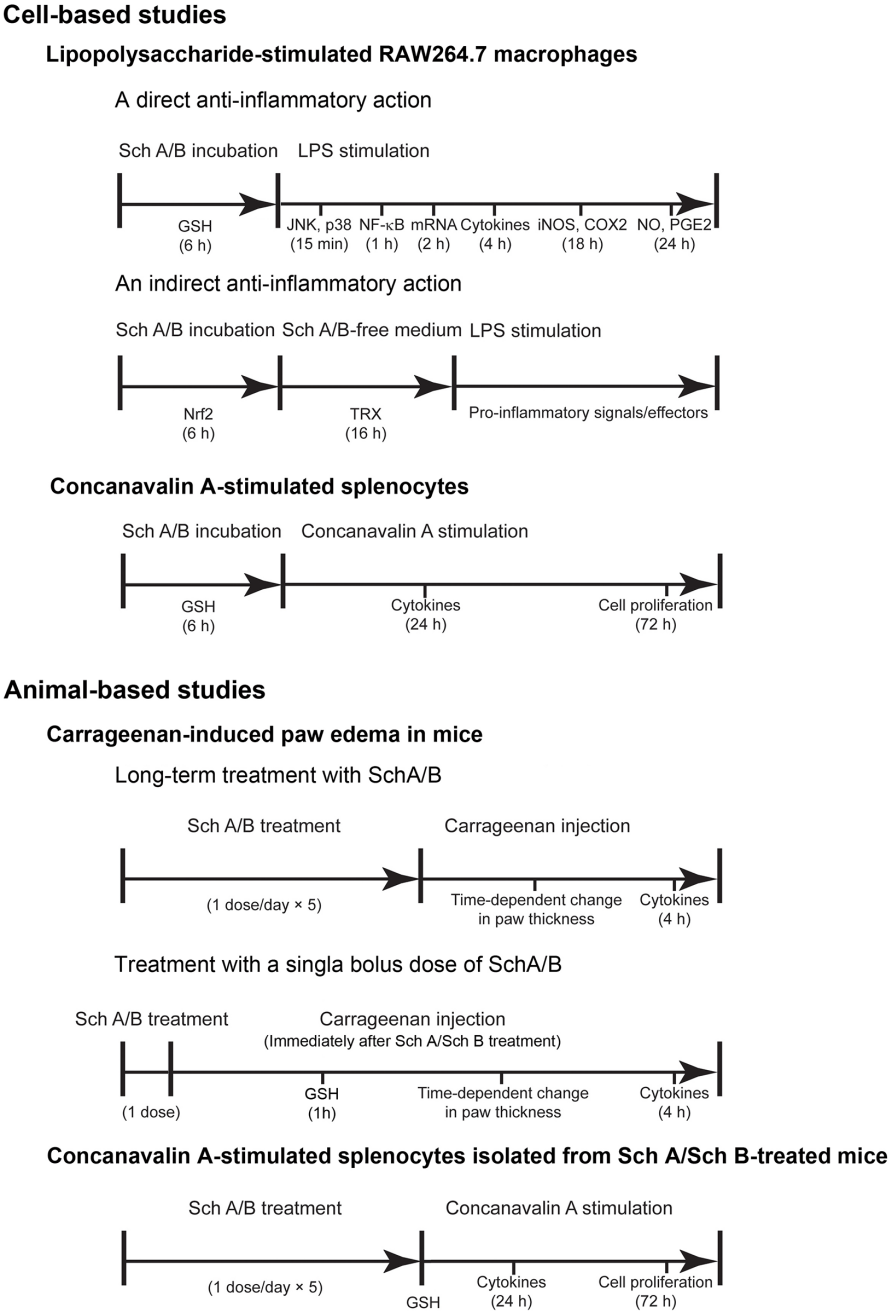
Experimental design of the present study.

For the investigation of the direct anti-inflammatory activities of Sch A and Sch B, macrophages were activated by LPS immediately following Sch A/Sch B incubation (Fig 3). In brief, RAW264.7 cells were incubated with Sch A (25 and 50 μM), Sch B (25 and 50 μM) and Sch A in combination with Sch B (Sch A+Sch B, 25 μM each) for 6 h. Sch A/Sch B-incubated cells were then exposed to LPS (1 μg/mL) for 1–24 hours and were harvested for biochemical analysis.

For the investigation of indirect anti-inflammatory activities of Sch A and Sch B, Sch A/Sch B-incubated macrophages were activated by LPS after an additional incubation for 16 h in tested compound-free medium for the induction of the antioxidant response (Fig 3). RAW264.7 cells were incubated with Sch A, Sch B and Sch A+Sch B for 6 h, followed by the incubation with for 16 h in the absence of the test compounds. Cells harvested after 6 h of incubation with Sch B were assessed for Nrf2 nuclear translocation, and cells harvested at 16 h post-Sch B exposure were analyzed for TRX levels. The incubated (16 h) cells were then exposed to LPS (1 μg/mL) for 1–24 hours and were harvested for biochemical analysis.

### Co-incubation with GST inhibitor, thiol/non-thiol antioxidants and cytochrome P450 (CYP) inhibitor

To delineate the involvement of various components in the anti-inflammatory actions of Sch A and Sch B, specific enzyme inhibitors and antioxidants were used. Quinidine (a GST inhibitor, 18 μM), N-acetyl cysteine (NAC, a thiol antioxidant, 5 mM), Trolox (a non-thiol antioxidant, 500 μM) and 1-aminobenzotriazole (ABT, a CYP inhibitor, 10 mM) were co-incubated with Sch A/Sch B in the LPS-activated macrophages.

### Knockdown Nrf2 expression by siRNA

Transfection with Nrf2 siRNA was performed according to the manufacturer’s instructions using the target sequence of sense, 5-UUAAGUGGCCCAAGUCUUGCUCCA-3; antisense, 5-UGGAGCAAGACU UGGGCCACUUAAA-3 for Nrf2 siRNA (Invitrogen, Eugene, OR, USA). Cells were transfected with Nrf2 siRNA (50 nmol/L) with Lipofectamin RNAiMAX transfection reagent according to the procedures modified by Chiu *et al*. [13]. The transfection efficiency of siRNA, as assessed by using BLOCK-iTTM Fluorescent Oligo, was found to be 95%.

### Sch A/Sch B incubation and Con A activation in lymphocytes

Isolated lymphocytes were incubated with Sch A (25 and 50 μM), Sch B (25 and 50 μM) and Sch A+Sch B (25 μM) for 6 h. Then, cells were harvested for the measurement of cellular GSH levels. After the 6 h incubation with Sch A/Sch B, Con A was added at final concentrations of 1, 2 and 4 μg/mL and further incubated for 24 or 72 h (Fig 3). The release of T helper cell 1 cytokines was measured at 24 h after the Con A incubation, and the extent of lymphocyte proliferation was determined colorimetrically by an MTT-based cell proliferation assay at 72 h post-Con A activation. The extent of lymphocyte proliferation was determined by measuring the absorbance at 600 nm using a Victor V3 Multi-label Counter. The stimulation index (SI) was calculated using the equation: SI = mean absorbance of cells stimulated with Con A/mean absorbance of cells not stimulated with Con A. The extent of Con A-stimulated proliferation of isolated lymphocytes was estimated by computing the area under the curve (AUC) of a graph plotting stimulatory indices against Con A concentration.

### Isolation of RNA and PT-PCR

To determine the expression of inflammatory effectors, macrophages were harvested at 2 h following NF-κB activation. For the determination of TRX expression, macrophages were harvested at 2 h after Nrf2 activation. Total RNA of the cells was extracted using a RNA purification kit (PureLink RNA Mini Kit) according to manufacturer’s instructions. The RNA yield was determined by the measurement of UV absorbance at 260 and 280 nm. The quality of RNA was assessed by the ratio of absorbance at 260 nm to absorbance at 280 nm (i.e. > 1.8). RNA (2.5 μg) was reversely transcribed into cDNA using a cDNA reverse transcription kit (SuperScript VILO Master Mix). An aliquot of 100 ng cDNA sample was subjected to real time polymerase chain reaction (rt-PCR) using custom-made Taqman Array Plates with a TaqMan PCR Master Mix in the Applied Biosystems 7500 Fast Real-Time PCR system (Life Technologies). The value of cycle threshold (CT value) was calculated. The CT values (which are indicative of relative cDNA levels) of target genes were normalized with the respective CT value of internal control (18S ribosomal RNA).

### Western blot analysis

The anti-JNK, anti-phospho JNK, anti-p38 and anti-phospho p38 primary antibodies as well as the horseradish peroxidase-conjugated secondary antibody were purchased from Cell Signaling Technology Inc. (Danvers, MA, USA). The anti-COX2, anti-Nrf2 and anti-TRX antibodies were purchased from purchased from Santa Cruz Biotechnology Inc. (Santa Cruz, CA, USA). The anti-iNOS antibody was purchased from Enzo Life Sciences (Lausen, Switzerland). The anti-β-actin antibody was purchased from Sigma-Aldrich Co (St. Louis, MO, USA). The anti-lamin B1 was purchased from Abcam, Inc. (Cambridge, MA, USA). The lysates of RAW264.7 cells were mixed with Laemmli’s loading buffer, boiled for 5 min, and separated by gel electrophoresis (SDS-PAGE) in 10% sodium dodecyl sulfate-polyacrylamide at 140 V followed by electroblotting to nitrocellulose membranes [purchased from Bio-Rad, (Hercules, CA, USA)] for 1.15 h at 100 V. Membranes were blocked for 1 h with 5% non-fat milk in Tris-buffered saline with Tween-20 (TBS-T, 50 mM Tris, 150 mM NaCl, 0.05% Tween-20) at room temperature and subsequently probed with anti-JNK (1:1000), anti-phospho JNK (1:1000), anti-p38 (1:1000), anti-phospho p38 (1:1000), anti-COX2 (1: 500), anti-iNOS (1:1000), anti-Nrf2 (1:1000) or TRX (1:500) overnight. The membranes were rinsed with TBS-T and incubated with a horseradish peroxidase-conjugated secondary antibody (1:5000). Following the incubation, the membranes were rinsed with TBS-T, and bound antibodies were detected by using enhanced chemiluminescence, following the manufacturer’s instructions (Cell Signaling Technology). The membranes were stripped and probed for anti-β-actin (1:5000) or anti-lamin B1 (1:1000) as loading controls. The molecular weight of the protein of interest was estimated using Blue Protein Standard marker, Broad Range (New England Biolabs Inc., Ipswich, U.S.A.). Individual band densities were quantified by Quantiscan.

### Enzyme-linked immunosorbent assay (ELISA)

The levels of TNF-α, IL-1β and IL-6, IL-2 in culture media or mouse paw tissue were measured using EILSA Kits (Life technologies, Grand Island, USA). The level of PGE2 in the culture medium from LPS-activated macrophages was measured by ELISA using an assay kit purchased from GE healthcare (Uppsala, Sweden). The levels of T helper cell 1 (Th1), T helper cell 2 (Th2) and T helper cell 17 (Th17) in the culture medium from the Con A-activated lymphocytes isolated from Sch A/Sch Btreated mice were measured by using mouse Th1/Th2/Th17 Cytokine Muti-analyte ELISArray Kits purchased from Qiagen Inc. (Valencia, CA, USA). All ELISAs were performed according to manufacturer’s instructions.

### Luciferase reporter gene assay

Nrf2-luciferase reporter, which consists of an Nrf2 responsive firefly luciferase construct and a constitutively expressing Renilla luciferase construct at a ratio of 40:1, was purchased from SABiosciences (Frederick, MD, USA). NF-κB-luciferase reporter, which consists of an NF-κB responsive firefly luciferase construct and a constitutively expressing Renilla luciferase construct at a ratio of 40:1, was purchased from SABiosciences. In brief, RAW264.7 cells were grown to 70–80% confluence in a 24-well cell culture dish and transfected with Nrf2-luciferase or NF-κB-luciferase reporter (0.5 μg/well) using Lipofectamine LTX & PLUS reagent according to the manufacturer's protocol. At twenty-four hours post-transfection, the transfected cells were incubated with Sch B as indicated previously. The incubated cells were washed with phosphate-buffered saline (PBS) and lysed with the buffer provided by the dual-luciferase reporter assay kit [Promega (Madison, WI, USA)]. Luciferase activities in the cell lysates were then measured using a Victor V3 Multi-label Counter. Renilla luciferase expression was used to normalize the various transfection efficiencies in the samples.

### Biochemical analyses

The activity of GST from cell lysate samples was measured using enzymatic methods as described in Chiu *et al*. [20]. The level of GSH was measured using the method of Griffith [21].

### Effects of Sch A and Sch B treatment on carrageenan-induced paw edema in mice

Female ICR mice were randomly divided into groups of 5–6 animals each. For the acute treatment, mice were given a single oral dose of Sch A (1 mmol/kg), Sch B (1 mmol/kg) and Sch A+Sch B (0.5 mmol/kg) while control mice received the vehicle (oil) only. Immediately after the Sch A or Sch B treatment, the left hind limb in ketamine chloride-anesthetized mice was injected with carrageenan (50 μL, 1%, w/v in sterile saline). The changes in paw thickness was measured using a digital caliper (Mitutoyo, Japan) and monitored for 4 h. Mice were then sacrificed by cardiac excision under ketamine chloride-induced anesthesia and paw tissue was obtained. Paw tissue was homogenized with 2.5 mL homogenizing buffer [10 mM 4-(2-hydroxyethyl)-1-piperazineethanesulfonic acid, 100 μM ethylenediaminetetraacetic acid, 0.32 M sucrose and proteinase inhibitor cocktails] using a tissue homogenizer ULTRA-TURRAX T25 (IKA laboratory technology, Wilmington, NC, USA) at 95,000 rpm and the cytosolic fraction was obtained by centrifugation at 10,000 ×*g* for 20 min at 4°C. Levels of cytokines and protein (for normalization) in the cytosolic fraction were measured. For the long-term treatment, mice were orally administered with Sch A (0.25 nmol/kg/d × 5 d), Sch B (0.25 nmol/kg/d × 5d) and Sch A+Sch B (0.125 nmol each/kg/d × 5d) while control mice received the vehicle (oil) only. One hour after the last dosing, paw edema was induced by carrageenan injection as described above.

### Effect of acute treatment with Sch A and Sch B on lymphocytes isolated from the blood of treated mice

Female ICR mice were randomly divided into groups of 5–6 animals each. Mice were given a single oral dose of Sch A (1 mmol/kg), Sch B (1 mmol/kg) and Sch A+Sch B (0.5 mmol/kg each) while control mice received the vehicle (oil) only. One hour after the Sch A/Sch B treatment, heparinized blood samples were drawn from ketamine-anesthetized mice by cardiac puncture. Mice were then sacrificed by cardiac excision under ketamine chloride-induced anesthesia. Lymphocytes were isolated by centrifugation in Histopaque 1083 at 400 ×*g* at room temperature for 30 min. The lymphocyte-rich opaque layer was collected and washed 3 times with PBS. The isolated lymphocytes were then lysed with 3% 5-sulfosalicylic acid. GSH levels of the samples were then measured.

### Effect of Sch A and Sch B treatment on con A-induced cell proliferation and release of cytokines in isolated lymphocytes from treated mice

Female ICR mice were randomly divided into different groups of 5–6 animals each. Mice were given Sch A (0.25 nmol/kg/d × 5d), Sch B (0.25 nmol/kg/d × 5d) and Sch A+Sch B (0.125 nmol each/kg/d × 5d) administered orally while control mice received the vehicle (oil) only. One hour after the last dosing, mice were then sacrificed by cardiac excision under ketamine chloride-induced anesthesia. Lymphocytes were isolated using the method described above. The cell suspension (5 × 10^6^ cell/mL) was activated with Con A (4 μg/mL, 24 h incubation) for the assessment of Th1/Th2/Th17 cytokine profiles or with Con A (1–4 μg/mL, 72 h incubation) for the examination of cell proliferation.

### Protein Assay

Protein concentrations of cell lysates were determined using a Bio-Rad protein assay kit (Hercules, CA, USA).

### Statistical analysis

Data were analyzed by *t*-test or one-way Analysis of Variance (ANOVA). Post-hoc multiple comparisons were performed using TUKEY. P values < 0.05 were regarded as statistically significant. All statistical analyses were performed using GraphPad Prism 3, SPSS 17.0 or Microsoft Office Excel 2013.

## Results

### Sch A/ Sch B causes activation of GST and depletion of cellular GSH in RAW264.7 macrophages

**Fig 4:**
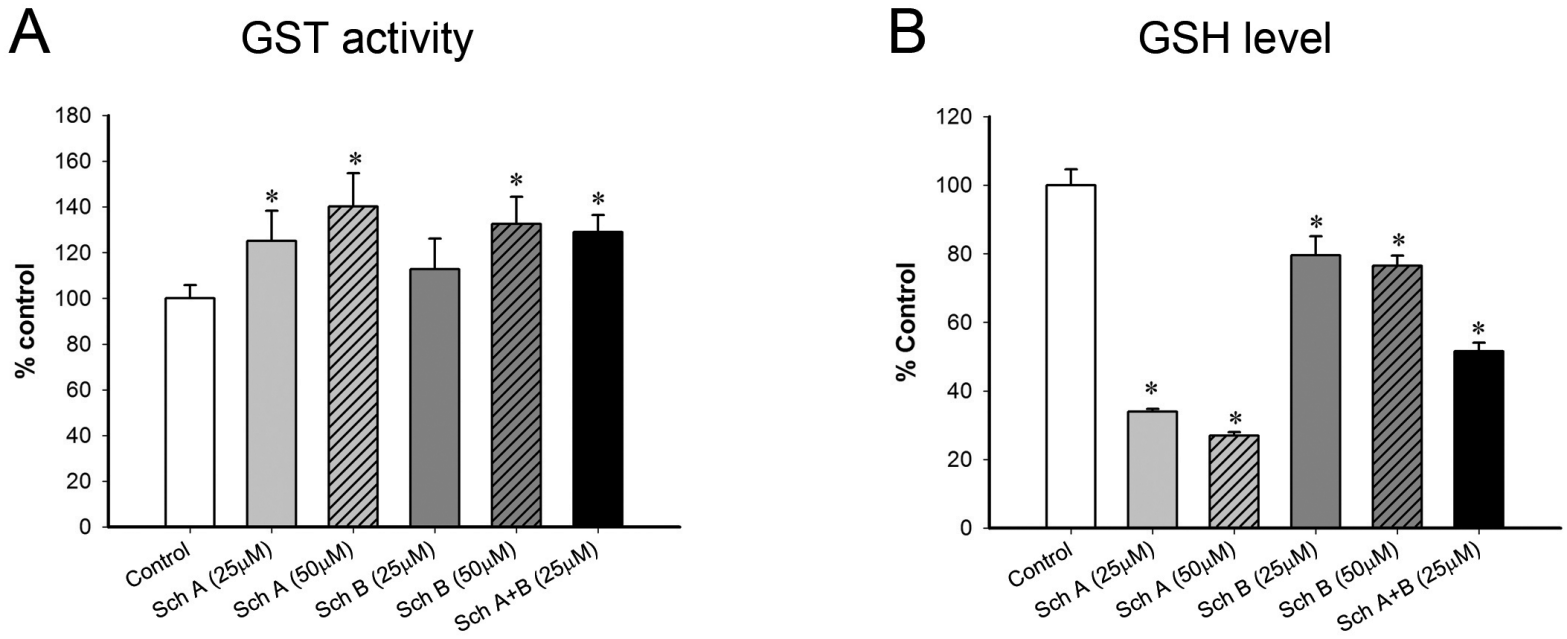
Effect of Sch A and Sch B incubation on glutathione S-transferase (GST) activity and reduced glutathione (GSH) levels in RAW264.7 macrophages. RAW264.7 macrophages were exposed to Sch A and Sch B [alone (25 and 50 μM) or in combination (25 μM)], as described in Materials and Methods. (A) After 1 h incubation, cells were harvested and lysed for the measurement of GST activity. (B) The cellular GSH level was measured at 6 hour post-incubation. Data expressed as % control, by normalizing relative to the value of control (vehicle). Value given are means ± SEM, with n = 6. * Significantly different from the control.

Incubation with Sch A or Sch B (25 and 50 μM) increased cellular GST activity in RAW264.7 macrophages, with the degree of stimulation caused by Sch A being greater (Sch A: 25 and 40% vs. Sch B: 13 and 32%, respectively) (Fig 4). The co-incubation with Sch A and Sch B (i.e. Sch A+Sch B, 25 μM each) also increased cellular GST activity (29%). The incubation with Sch A or Sch B (25 and 50 μM) also caused a decrease of cellular GSH in a concentration-dependent manner, with the extent of depletion induced by Sch A being more prominent (Sch A: 66 and 73% vs. Sch B: 20 and 23%) (Fig 4). The co-incubation with Sch A and Sch B depleted cellular GSH by 48%

### Sch A/ Sch B suppresses pro-inflammatory signaling pathways, pro-inflammatory cytokines and associated inflammatory effectors in LPS-stimulated macrophages

**Fig 5:**
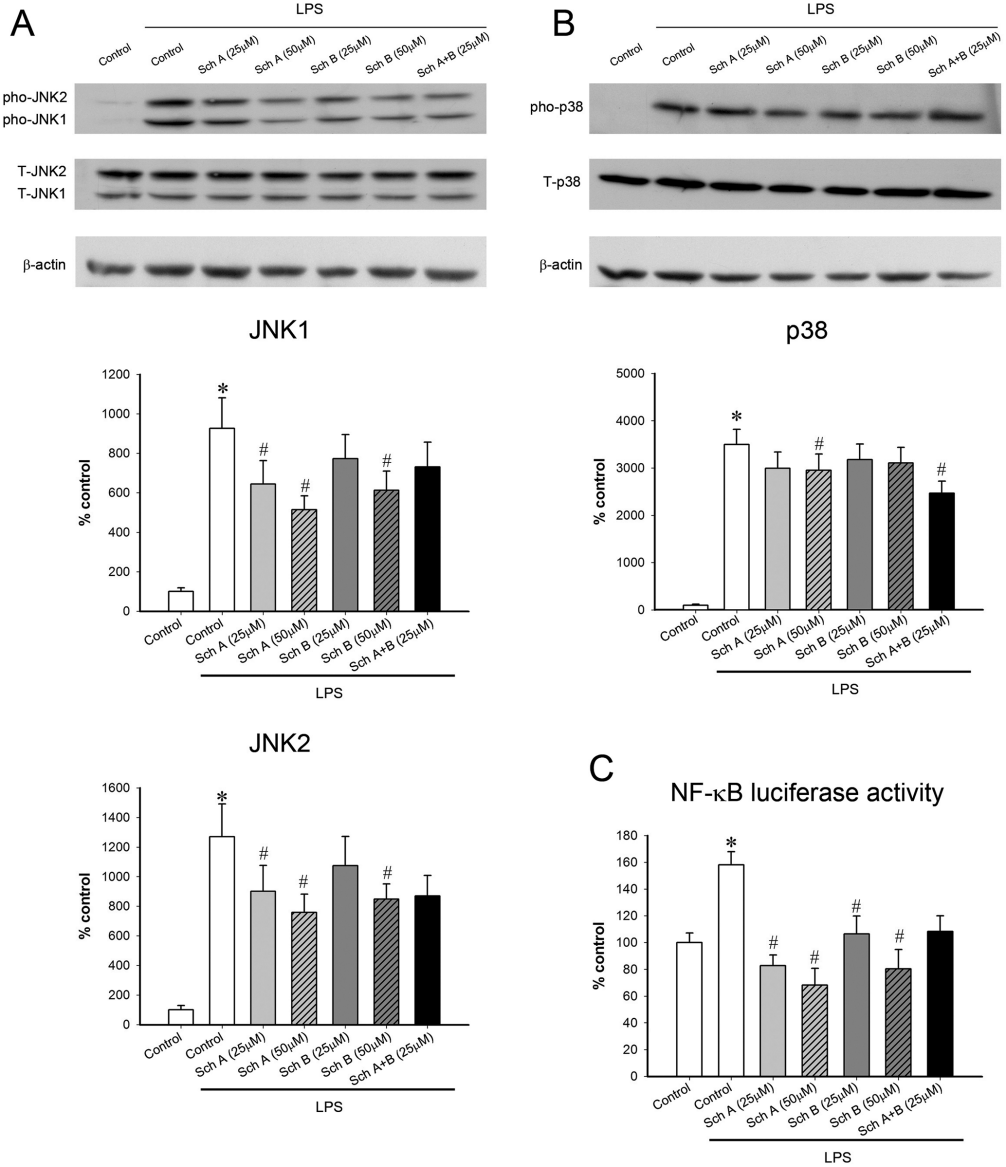
Effect of a 6-h incubation with Sch A and Sch B in lipopolysaccharide (LPS)-activated pro-inflammatory signals in RAW264.7 macrophages. After incubation with Sch A and Sch B for 6 h, RAW264.7 macrophages were challenged with LPS (1 μg/mL) for 15 min. (A, B) The levels of phospho-JNK1/2 (and phospho-p38) and total-JNK1/2 (and total p38) were measured using Western blot analysis. The ratio of phoshos-JNK1/2 to total-JNK1/2 as well as the ratio of phospho-p38 to total p38 (which are indicative of JNK1/2 and p38 activation) were estimated and expressed as % control, by normalizing with the value of the non-LPS group (JNK1/2: left panel, p38: right; upper panel). (C) After transfection with NF-κB-luciferase reporter in RAW264.7 macrophages, the RAW264.7 cells were incubated with Sch A or Sch B, as described in Materials and Methods. NF-κB-luciferase reporter, which consists of an NF-κB- responsive firefly luciferase construct and a constitutively expressed Renilla luciferase. Following exposure to LPS for 1 h, luciferase activities in the cell lysates were measured. Renilla luciferase expression was used to normalize the varying transfection efficiencies among the samples. NF-κB reporter activity was expressed as % control, by normalizing with the value of the non-LPS control. Value given are means ± SEM, with n = 3. * Significantly different from the non-LPS control; # significantly different from the LPS control.

**Fig 6:**
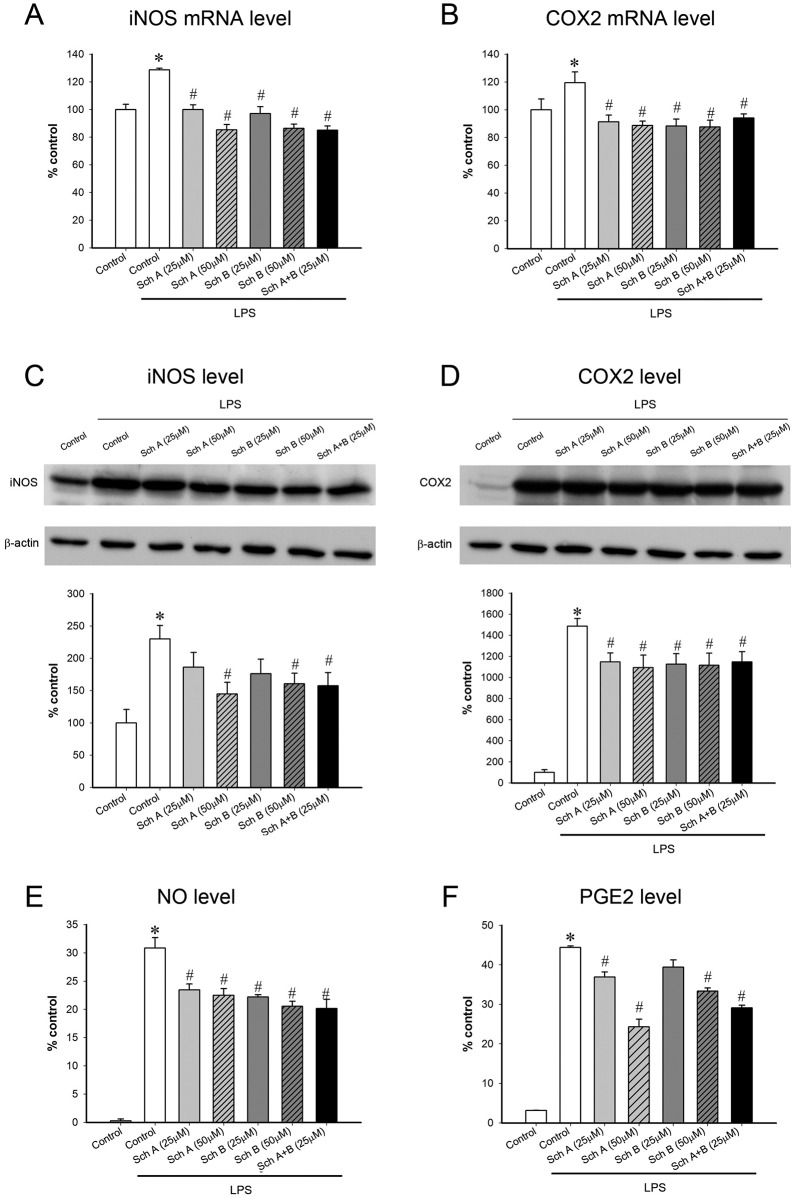
Effect of a 6-h incubation with Sch A and Sch B in lipopolysaccharide (LPS)-activated pro-inflammatory effectors in RAW264.7 macrophages. (A, B) Following incubation with Sch A or Sch B, RAW264.7 macrophages were exposed to LPS for 2 h. RNA of the cell lysate was isolated using a RNA isolation kit. The isolated RNA was converted into cDNA using a cDNA synthesis kit. The cDNA levels of inducible nitric oxide synthase (iNOS) and cyclooxygenase 2 (COX2) were measured by real time polymerase chain reaction using TaqMan Array plates on the 7500 Fast system. The value of cycle threshold (CT value) was calculated. The CT values (which are indicative of relative cDNA levels) of iNOS and COX2 were normalized with the respective CT value of internal control (18S ribosomal RNA). Data are expressed as % non-LPS control by normalizing relataive to the value of non-LPS stimulated cells (Upper panel). (C, D) Following exposure to LPS for 18 h, the levels of iNOS and COX2 were measured using Western blot analysis. The levels of iNOS and COX2 were normalized relative to β-actin and expressed as % non-LPS control (middle panel). (E, F) Following exposure to LPS for 24 h, the levels of nitric oxide (NO, the product of an iNOS-catalyzed reaction) and prostaglandin E2 (PGE2, the product of a COX2-catalyzed reaction) were measured by Griess assay and ELISA, respectively. Data are expressed as % non-LPS control by normalizing relative to non-LPS stimulated cells (lower panel). Values given are means ± SEM, with n = 3–5. * Significantly different from the non-LPS control; # significantly different from the LPS control.

LPS activated the pro-inflammatory signaling cascade, as evidenced by increases in the expression of JNK1 (8.2 fold), JNK2 (11.7 fold), p38 (33.9 fold) and NF-κB (58%) (Fig 5). The activation of the pro-inflammatory signaling pathway caused by LPS was associated with increased levels of TNF-α (9700 fold), IL-1β (12.1 fold), IL-6 (146 fold), iNOS (1.2 fold), NO (117 fold), COX2 (13.3 fold) and PGE2 (14.1 fold) in challenged macrophages (Fig 6 and Table 1). While Sch A or Sch B incubation (25 μM) inhibited the pro-inflammatory signal cascade and inflammatory effectors to varying degrees, the incubation with Sch A or Sch B (50 μM) invariably suppressed the inflammatory indices (Figs 5 and 6). Incubation with Sch A or Sch B (50 μM) suppressed the extent of LPS-induced activation of JNK1 (50 and 38%), JNK2 (44 and 36%) and NF-κB (138 and 98%). Only Sch A (but not Sch B) incubation inhibited the activation of p38 in LPS-activated macrophages (by 16%). Incubation with Sch A or Sch B (50 μM) reduced the extent of release of TNF-α (48 and 48%), IL-1β (66 and 55%) and IL-6 (41 and 27%) in LPS-activated macrophages. Sch A or Sch B incubation (50 μM) reduced the extent of LPS-induced increase in mRNA levels of iNOS (151 and 148%), the concentration of iNOS protein (57 and 42%) and the level of NO (27 and 34%), as well as mRNA contents of COX2 (158 and 164%), the amount of COX2 protein (16 and 21%) and the level of PGE2 (49 and 27%).

**Table 1:** Effect of 6-h incubation with Sch A and Sch B in lipopolysaccharide (LPS)-activated pro-inflammatory cytokines in RAW264.7 macrophages.

Cytokine (pg/mg protein)	TNF-α	IL-1β	IL-6
Non-LPS			
Control	1.53 ± 0.12	1.14 ± 0.17	0.77 ± 0.52
LPS			
Control	14860 ± 1350*	14.96 ± 2.24*	113.73 ± 9.93*
Sch A 25μM	8667 ± 1530^#^	7.76 ± 0.51^#^	55.06 ± 5.59^#^
Sch A 50μM	7717 ± 621^#^	5.79 ± 1.37^#^	67.64 ± 5.67^#^
Sch B 25μM	13220 ± 960	8.55 ± 0.19^#^	97.95 ± 4.51
Sch B 50μM	7676 ± 640^#^	6.19 ± 1.03^#^	83.26 ± 2.57^#^
Sch A+Sch B25μM	8349 ± 725^#^	7.31 ± 1.92^#^	63.78 ± 7.0^#^

TNF-α, tumor necrosis factor α; IL-1β, interleukin 1β; IL-6, interleukin 6.

* Significantly different from the non-LPS control;

^#^ significantly different from the LPS control.

### Effects of GST inhibition, and thiol and non-thiol antioxidants on the anti-inflammatory action produced by Sch A or Sch B in LPS-activated macrophages

To investigate the possible involvement of GST in the anti-inflammatory action produced by Sch A or Sch B incubation, the effects of quinidine (a GST inhibitor, 18 μM) on Sch A/Sch B-pre-incubated and LPS-activated macrophages were examined (Table 2). Quinidine completely abrogated the activation of GST, but it only slightly attenuated the depletion of GSH in Sch A/Sch B-incubated macrophages. Quinidine also suppressed the anti-inflammatory action of Sch A and Sch B, as indicated by a reversal in the changes in the levels of iNOS and NO in LPS-activated macrophages. To investigate the causal relationship between the anti-inflammatory action and GSH depletion in Sch A/Sch B-incubated macrophages, the effects of thiol and non-thiol antioxidants on Sch A/Sch B-incubated and LPS-activated macrophages were also examined. NAC (a thiol antioxidant, 5 mM) restored GSH levels after Sch A/Sch B incubation (50 μM alone or 25 μM in combination). The restoration of GSH by NAC was associated with an attenuation of the anti-inflammatory action produced by Sch A or Sch B, as evidenced by the reversal of changes in levels of iNOS and NO. Trolox (a non-thiol antioxidant, 500 μM) partially prevented the GSH depletion caused by Sch A (but not Sch B or Sch A+Sch B). In addition, Trolox did not attenuate the anti-inflammatory action of Sch A, Sch B or Sch A+Sch B.

**Table 2:** Effects of GST inhibitor and thiol/non-thiol antioxidants on the anti-inflammatory actions produced by Sch A and Sch B in lipopolysaccharide-activated RAW264.7 macrophages.

	GST inhibitor		Thiol antioxidant		non-Thiol antioxidant	
	Non-Quinidine	Quinidine (18 μM)	Non-NAC	NAC (5 mM)	Non-Trolox	Trolox (500 μM)
GST (% change vs respective control)						
Sch A 50 μM	↑(28%)	No Sig. Change	N/A		N/A	
Sch B 50 μM	↑(15%)	No Sig. Change				
Sch A + Sch B 25 μM	↑(18%)	No Sig. Change				
GSH (% change vs respective control)						
Sch A 50 μM	↓(68%)	↓(61%)	↓(67%)	↓(32%)	↓(66%)	↓(40%)
Sch B 50 μM	↓(25%)	↓(18%)	↓(12%)	↓(9%)	↓(21%)	↑(38%)
Sch A + Sch B 25 μM	↓(41%)	↓(38%)	↓(33%)	↓(23%)	↓(41%)	No Sig. Change
iNOS (% suppression vs LPS-control)						
Sch A 50 μM	75%	No Sig. suppress	32%	No Sig. suppress	42%	47%
Sch B 50 μM	44%	No Sig. suppress	41%	No Sig. suppress	54%	24%
Sch A + Sch B 25 μM	38%	No Sig. suppress	32%	No Sig. suppress	63%	53%
NO (% suppression vs LPS-control)						
Sch A 50 μM	31%	No Sig. suppress	35%	No Sig. suppress	32%	45%
Sch B 50 μM	20%	No Sig. suppress	15%	No Sig. suppress	21%	43%
Sch A + Sch B 25 μM	25%	No Sig. suppress	28%	No Sig. suppress	28%	41%

GST, glutathione S-transferase; NAC, N-acetyl cysteine; GSH, reduced glutathione; iNOS, inducible nitric oxide synthase; NO, nitric oxide. No significant change was indicated as No Sig. Change; No significant suppression was indicated as No Sig. suppress.

### Sch B activates Nrf2 and induces the expression of TRX in macrophages

**Fig 7:**
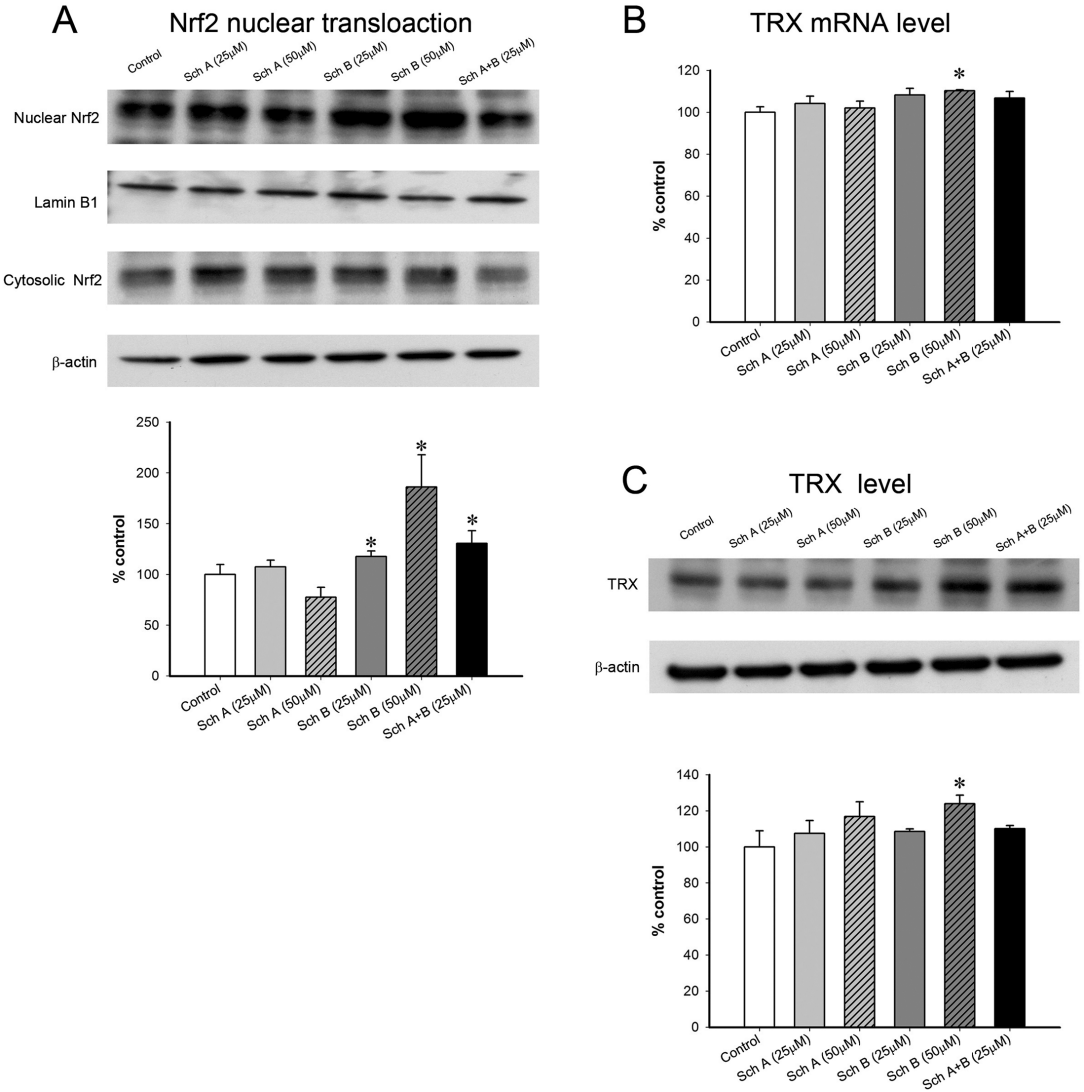
Effect of Sch A and Sch B incubation on the antioxidant response in RAW264.7 macrophages. RAW264.7 macrophages were incubated with Sch A or Sch B for 6 h. (A) Nuclear factor erythroid 2-related factor 2 (Nrf2) is a master transcription factor for the antioxidant system. Levels of Nrf2 and β-actin in cytosolic fractions as well as Nrf2 and lamin B1 in nuclear fractions of macrophages were measured using Western blot analysis. Whereas the level of Nrf2cytosolic was normalized relative to the β-actincytosolic level, Nrf2nuclear content was normalized relative to the lamin-B1nuclear level. The ratio of normalized nuclear Nrf2 to normalized cytosolic Nrf2 was determined to estimate the extent of Nrf2 nuclear translocation (which is indicative of Nrf2 activation) in RAW264.7 macrophages (left panel). Data are expressed as % control by normalizing relative to the vehicle-incubated control group. (B) Following 2 h of-exposure to Sch A or Sch B, the mRNA level of thioredoxin (TRX) was measured, as described in Fig 6. (C) Following a 16 h-exposure to Sch A and Sch B, the level of TRX was measured using Western blot analysis. The amount of TRX was normalized relative to the β-actin level and expressed as % control (right; lower panel). Value given are means ± SEM, with n = 3. * Significantly different from the vehicle control.

Incubation with Sch B (25 and 50 μM), but not Sch A, activated Nrf2, as indicated by an increase in nuclear translocation of Nrf2 (18 and 85%) (Fig 7). Only Sch B incubation (50 μM) was found to significantly increase the levels of mRNA (4 h post-Sch B exposure) and protein of TRX (16 h-post Sch B exposure) by 10 and 30%, respectively (Fig 7).

### Sch B suppresses pro-inflammatory cytokines and inflammatory effectors in LPS-stimulated macrophages at 16 h-post exposure

**Fig 8:**
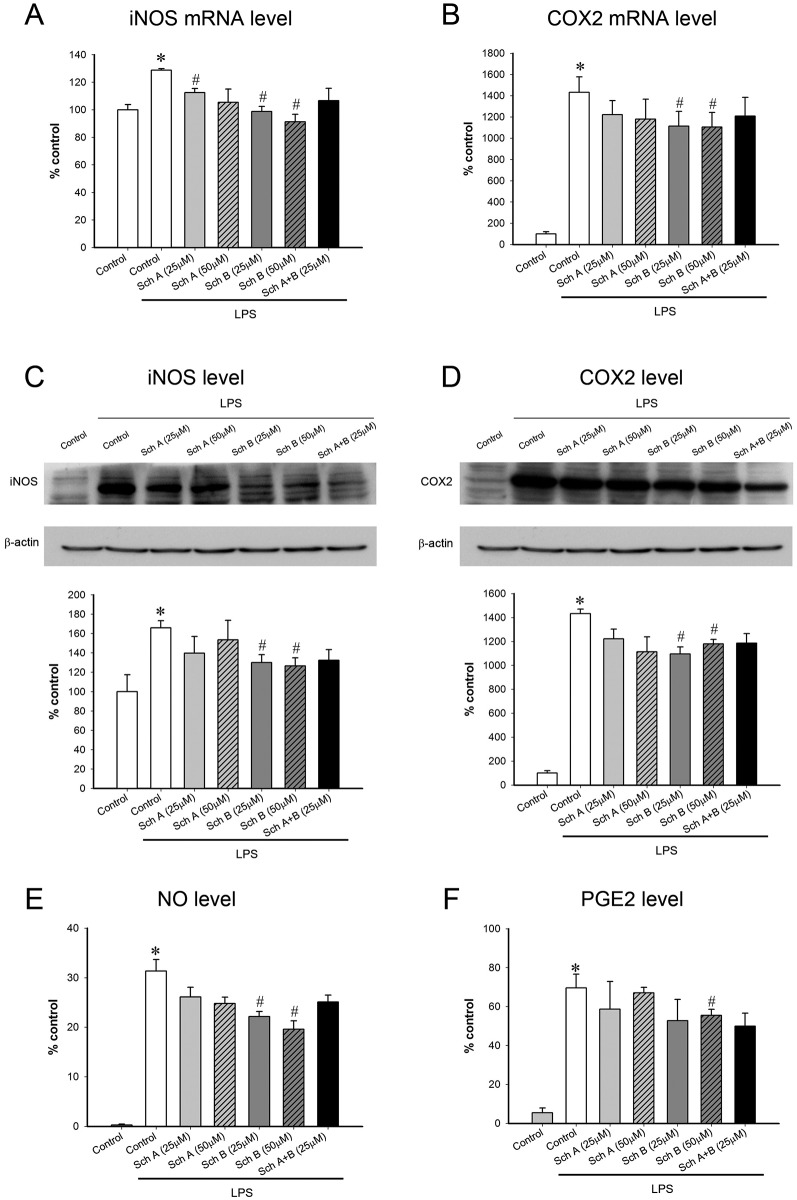
Effect of Sch B incubation on lipopolysaccharide (LPS)-activated pro-inflammatory effectors at 16 h-post exposure in RAW264.7 macrophages. No significant change was indicated as No Sig. Change; No significant suppression was indicated as No Sig. suppressAfter incubation with Sch A or Sch B for 6 h, RAW264.7 macrophages were further incubated with tested compound-free medium for 16 h for the induction of thioredoxin. Cells were then exposed to LPS for 2 h. (A, B) RNA of the cell lysate was isolated and cDNA levels of inducible nitric oxide synthase (iNOS) and cyclooxygenase 2 (COX2) were measured by real time polymerase chain reaction using TaqMan Array plates on the 7500 Fast system. The data were quantified as described in Fig 5. Data were expressed as % non-LPS control by normalizing with the value of non-LPS stimulated cells (Upper panel). (C, D) After exposure to LPS for 18 hours, the level of iNOS and COX2 was measured using Western blot analysis. The amount of iNOS and COX2 were normalized with respective β-actin level and expressed as % non-LPS control (middle panel). (E, F) After exposure to LPS for 24 h, the levels of nitric oxide (NO, the product of iNOS-catalyzed reaction) and prostaglandin E2 (PGE2, the product of COX2-catalyzed reaction) were measured by Griess assay and ELISA, respectively. Data were expressed as % non-LPS control by normalizing with the value of non-LPS stimulated cells (lower panel). Value given are means ± SEM, with n = 3–5. * Significantly different from the non-LPS control; # significantly different from the LPS control.

Incubation with Sch A, Sch B or Sch A+Sch B did not produce detectable changes in the pro-inflammatory signaling cascade, as assessed by the activation of JNK1, JNK2, p38 and NF-κB, at 16 h-post exposure in LPS-activated macrophages (data not shown). Only incubation with Sch B (but not Sch A and Sch A+Sch B) produced an anti-inflammatory action in LPS-activated macrophages. While the incubation with Sch B at 25 μM differentially attenuated the tested inflammatory parameters, the incubation with Sch B at 50 μM consistently suppressed all tested pro-inflammatory cytokines [TNF-α (40%), IL-1β (84%) and IL-6 (55%)] (Table 3) and inflammatory effectors [iNOS (60%), NO (38%), COX2 (25%) and PGE2 (27%)] (Fig 8). Sch A+Sch B incubation suppressed the tested inflammatory parameters to varying extents.

**Table 3:** Effect of Sch B incubation on lipopolysaccharide (LPS)-activated pro-inflammatory cytokines at 16 h- post Sch exposure in RAW264.7 macrophages.

Cytokine (pg/mg protein)	TNF-α	IL-1β	IL-6
Non-LPS			
Control	2.08 ± 0.52	2.04 ± 0.65	0.81 ± 0.04
LPS			
Control	18599 ± 2077*	7.76 ± 1.83*	95.94 ± 14.25*
Sch A 25μM	17781 ± 1090	7.89 ± 2.1	97.87 ± 14.25
Sch A 50μM	16899 ± 3688	6.74 ± 2.72	82.69 ± 10.21
Sch B 25μM	12017 ± 947^#^	2.35 ±0.51^#^	73.40 ± 7.7^#^
Sch B 50μM	11224 ± 2113^#^	2.95 ± 0.63^#^	43.5 ± 1.76^#^
Sch A+Sch B25μM	19274 ± 3098	1.98 ± 0.39^#^	81.13 ± 11.76

TNF-α, tumor necrosis factor α; IL-1β, interleukin 1β; IL-6, interleukin 6.

* Significantly different from the non-LPS control;

^#^ significantly different from the LPS control.

### Effects of CYP inhibitor and Nrf2 knockdown on the anti-inflammatory action produced by Sch B at 16 h post-exposure in LPS-activated macrophages

Earlier studies in our laboratory have demonstrated that Sch B can induce an Nrf2-driven antioxidant response via the CYP-catalyzed production of signaling ROS in various cell lines. In this regard, we investigated the involvement of CYP in the anti-inflammatory action of Sch B using a CYP inhibitor (Table 4). ABT (a CYP inhibitor, 10 mM) completely suppressed the Sch B-induced activation of Nrf2 and expression of TRX in macrophages. The abrogation of Sch B-induced activation of Nrf2 and expression of TRX was found to be associated with an attenuation of the Sch B-induced anti-inflammatory action, as evidenced by a reversal of changes in the levels of iNOS and NO in LPS-activated macrophages. The important role of Nrf2 activation in Sch B-induced anti-inflammatory action was also confirmed in Nrf2 knockdown macrophages (Table 4). The transfection with siRNA of Nrf2 suppressed the Sch B-induced expression of TRX in macrophages. The Sch B-induced anti-inflammatory action was also inhibited in siRNA of Nrf2-transfected macrophages, as indicated by a reversal of changes in the levels of iNOS and NO.

**Table 4:** Effects of CYP inhibitor and Nrf2 knock down on the anti-inflammatory action afforded by Sch B in lipopolysaccharide (LPS)-activated RAW264.7 macrophages.

	CYP inhibitor		Nrf2 knock down	
	Non-ABT	ABT (10 mM)	Non-siRNA Nrf2	siRNA Nrf2
Nrf2 luciferase activity (% change VS respective control)				
Sch A 50 μM	No Sig. Change	No Sig. Change	N/A	
Sch B 50 μM	↑(80%)	No Sig. Change	↑(60%)	No Sig. Change
Sch A + Sch B 25 μM	↑(32%)	No Sig. Change	N/A	
TRX (% change vs respective control)				
Sch A 50 μM	No Sig. Change	No Sig. Change	No Sig. Change	No Sig. Change
Sch B 50 μM	↑(35%)	No Sig. Change	↑(61%)	No Sig. Change
Sch A + Sch B 25 μM	No Sig. Change	No Sig. Change	No Sig. Change	No Sig. Change
iNOS (% suppression vs LPS-control)				
Sch A 50 μM	No Sig. suppress	No Sig. suppress	No Sig. suppress	No Sig. suppress
Sch B 50 μM	29%	No Sig. suppress	59%	No Sig. suppress
Sch A + Sch B 25 μM	38%	No Sig. suppress	No Sig. suppress	No Sig. suppress
NO (% suppression vs LPS-control)				
Sch A 50 μM	12%	No Sig. suppress	No Sig. suppress	No Sig. suppress
Sch B 50 μM	54%	29%	36%	16%
Sch A + Sch B 25 μM	39%	No Sig. suppress	No Sig. suppress	No Sig. suppress

CYP, cytochrome P450; ABT, 1-amimobenzotriazole; Nrf2, nuclear factor (erythroid-derived 2)-like 2; TRX, thioredoxin; iNOS, inducible, NO nitric oxide. No significant change was indicated as No Sig. Change; No significant suppression was indicated as No Sig. suppress.

### Sch A or Sch B causes depletion of GSH and suppresses the Con A-induced release of IL-2 as well as cell proliferation in isolated mouse splenocytes

**Fig 9:**
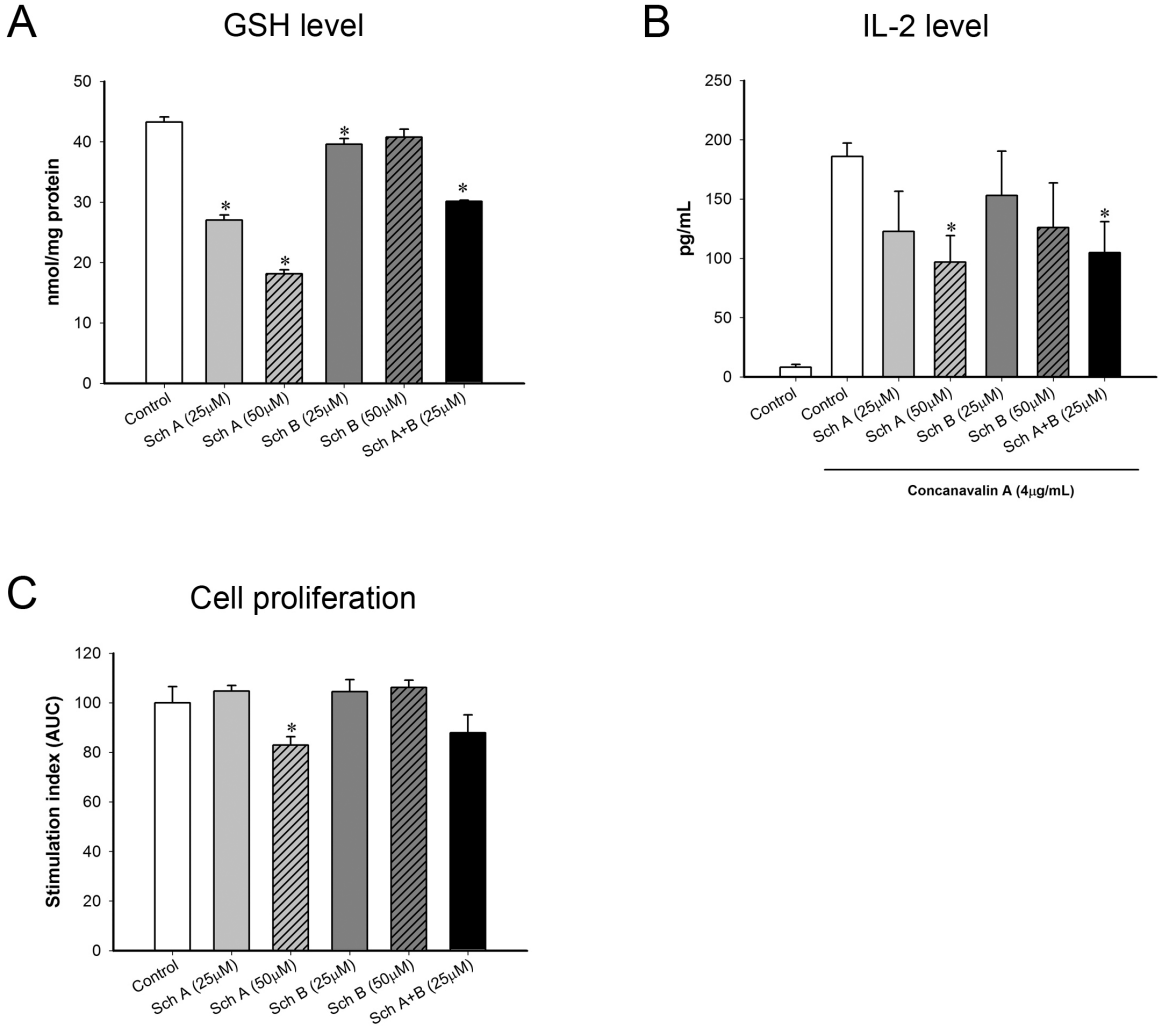
Effect of Sch A and Sch B incubation on levels of GSH and interleukin 2 (IL-2) and cell proliferation in concanavalin A (Con A)-activated splenocytes. Splenocytes were isolated from the spleens of female ICR mice as described in Materials and Methods. Isolated splenocytes were exposed to Sch A or Sch B [alone (25 and 50 μM) or in combination (25 μM)] for 6 h. (A) Cellular GSH levels were measured. Data are expressed as nmol/mg protein (left; upper panel). * Significantly different from the control group. The incubated cells were then stimulated with concanavalin A (4 μg/mL). (B) At 24 h after exposure to Con A, levels of IL-2 (in the culture medium) were measured using ELISA. Data are expressed in pg/mL (left; lower panel). Values given are means ± SEM, with n = 3–5. * Significantly different from the control group; # significantly different from the control group with LPS stimulation. (C) At 72 h after exposure to Con A (1, 2 and 4 μg/mL), the Con A-induced proliferation of isolated lymphocytes was measured using the MTT assay, as described in Materials and Methods. The area under the curve (AUC) plotting the absorbance at 570 nm against concentration of Con A was calculated and expressed as stimulation index. Data are expressed as % control by normalizing relative to the vehicle control group (right panel). Values given are means ± SEM, with n = 3–5. * Significantly different from the vehicle control group.

Incubation with Sch A concentration-dependently decreased cellular GSH levels in isolated mouse splenocytes (37 and 58%, at 25 and 50 μM, respectively) (Fig 9). Incubation with Sch B (25 μM) also slightly but significantly decreased the GSH level (8%). Sch A+ Sch B also decreased the GSH level, with the extent of depletion being 30%. The extent of GSH depletion caused by Sch A (50 μM) was paralleled by the suppressions of IL-2 release (48%) and cell proliferation (17%) in Con A-activated splenocytes (Fig 9). Despite the fact that Sch A+Sch B significantly suppressed the release of IL-2 (44%) in Con A-activated splenocytes, Sch A+Sch B did not significantly inhibit Con A-stimulated cell proliferation.

### Effects of acute and long-term treatment with Sch A or Sch B on carrageenan-induced paw edema in mice

**Fig 10:**
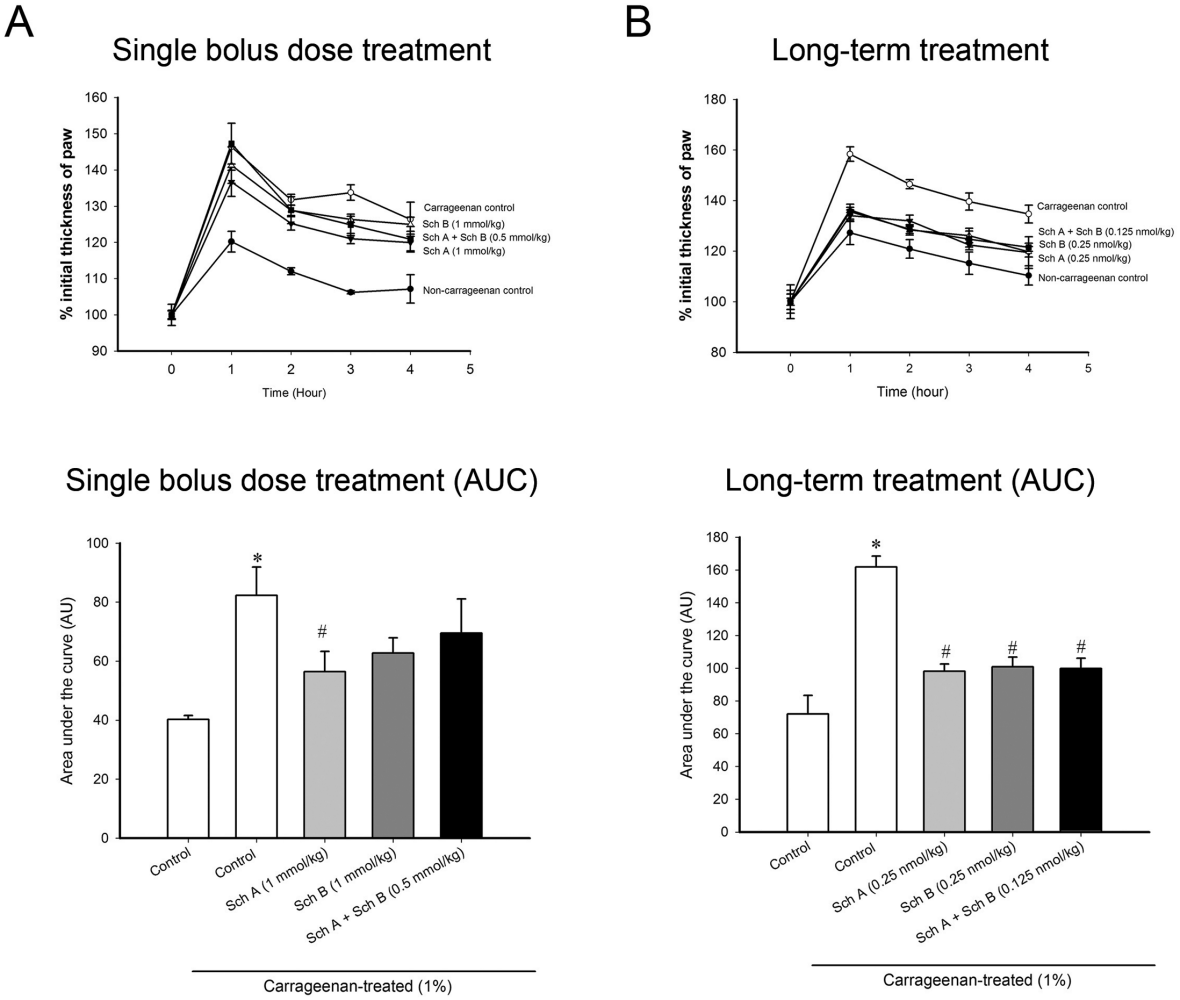
Effects of acute/long-term treatment with Sch A and Sch B in carrageenan-induced paw edema in ICR mice. (A, B) Female ICR mice were administered Sch A or Sch B [single oral bolus dose: alone (1 mmol/kg) or in combination (0.5 mmol/kg); long-term treatment: alone (0.25 nmol/kg/d) or in combination (0.125 nmol/kg/d) × 5 d], as described in Materials and Methods. The left hind limb of the Sch A/Sch Btreated mice was injected with carrageenan (50 μL, 1%, w/v in sterile saline). Time-dependent changes in the thickness of the paw were monitored (upper panel). The area under the curve (AUC) was calculated by plotting % initial thickness of paw against time (lower panel of A and B). Data are expressed as % control by normalizing with the value of vehicle control group. Value given are means ± SEM, with n = 5. * Significantly different from the vehicle control group.

**Fig 11:**
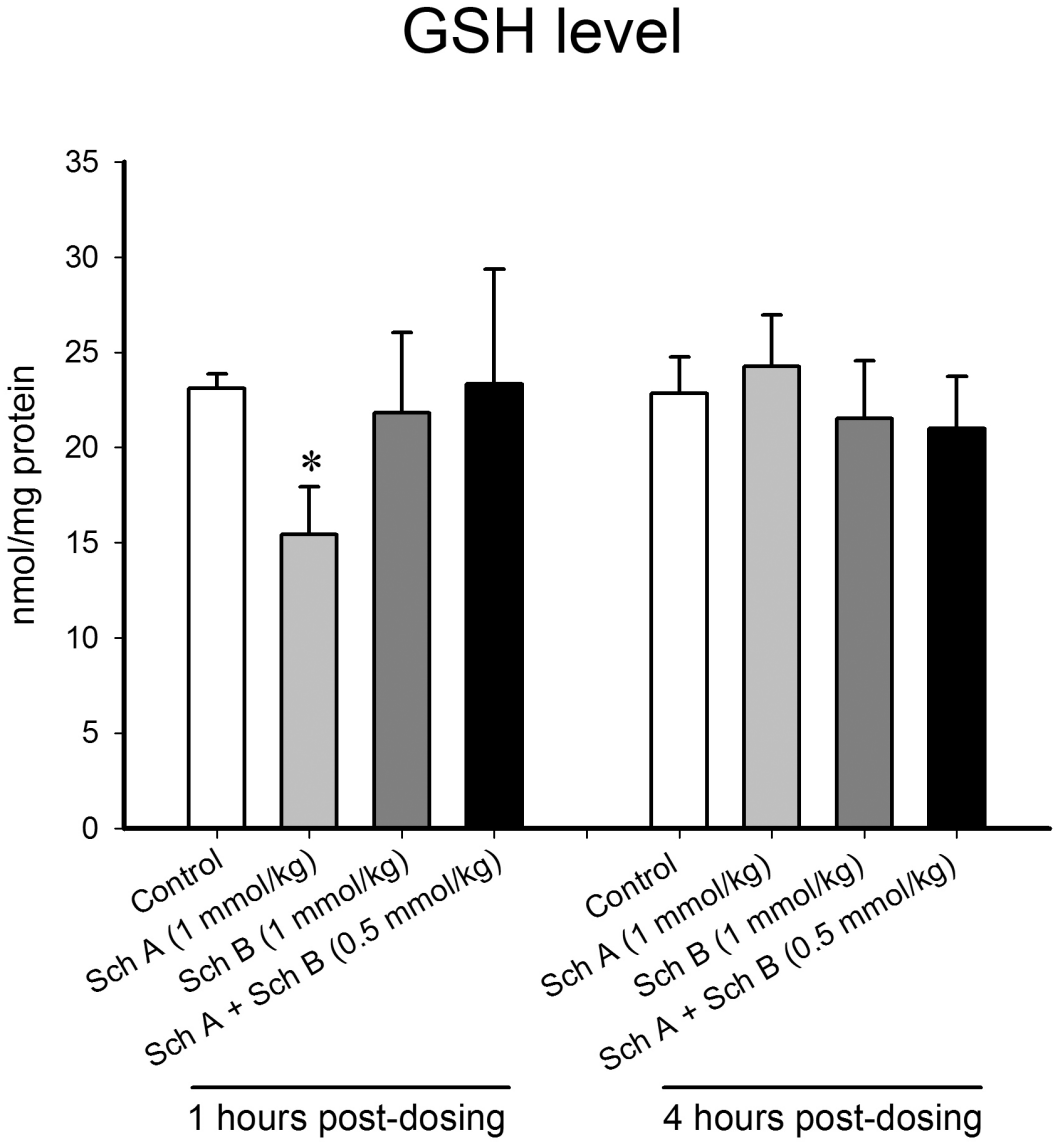
Effect of acute treatment with Sch A and Sch B on the level of GSH in lymphocytes isolated from ICR mice. Female ICR mice were administered Sch A or Sch B [single oral bolus dose: alone (1 mmol/kg) or in combination (0.5 mmol/kg)], as described in Materials and Methods. One h after the Sch A/Sch B treatment, heparinized blood samples were drawn from ketamine-anesthetized mice by cardiac puncture. Lymphocytes were isolated by centrifugation in Histopaque 1083, as described in Materials and Methods. Lymphocytes were washed with phosphate-buffered saline (PBS) and lysed with 3% SSA. GSH levels of isolated lymphocytes were measured. Value given are means ± SEM, with n = 5–6. * Significantly different from the vehicle control group.

To extend the results obtained from cell-based studies, the anti-inflammatory actions of Sch A and Sch B were examined in a mouse model of inflammation—namely, carrageenan-induced paw edema. Carrageenan injection (1% w/v in sterile saline) into the paws of mice caused edema (by 104%), when compared with vehicle-injected mice. Treatment with a single bolus dose of Sch A (1 mmol/kg), but not Sch B (1 mmol/kg) or Sch A+Sch B (0.5 mmol/kg each), significantly suppressed paw edema in carrageenan-injected mice, with the degree of inhibition being 31% (Fig 10). The suppression of paw edema produced by acute Sch A treatment in carrageenan-injected mice was found to be associated with decreases in the release of IL-1β (45%) and IL-6 (41%) in paw tissue (Table 5). Depletion of GSH (33%) in lymphocytes isolated from Sch A-treated mice at 1 hour post-dosing was observed (Fig 11). On the other hand, long-term treatment with Sch A (0.25 nmol/kg/d × 5d), Sch B (0.25 nmol/kg/d × 5d) or Sch A+Sch B (0.125 nmol/kg/d × 5d) reduced the extent of paw edema, with the degree of suppression being 39, 38 and 38%, respectively (Fig 10). The suppression of paw edema afforded by long-term treatment with Sch A, Sch B and Sch A+Sch B was found to be associated with a parallel inhibition of IL-1β release (by 45, 48 and 37%, respectively) in the paw tissue (Table 6).

**Table 5:** Effect of the acute treatment with Sch A and Sch B on pro-inflammatory cytokines in carrageenan-induced paw edema in mice.

Cytokine (pg/mg protein)	IL-1β	IL-6
Non-Carrageenan		
Control	15.15 ± 4.41	5.39 ± 0.89
Carrageenan		
Control	32.32 ± 2.56*	76.71 ± 14.35*
Sch A 1 mmol/kg	17.62 ±2.59^#^	45.54 ± 7.24^#^
Sch B 1 mmol/kg	26.86 ± 4.59	63.56 ± 13.56
Sch A+Sch B 0.5 mmol/kg	25.14 ± 4.94	67.07 ± 10.59

IL-1β, interleukin 1β; IL-6, interleukin 6.

* Significantly different from the non-carrageenan control;

^#^ significantly different from the carrageenan control.

**Table 6:** Effect of the long-term treatment with Sch A and Sch B on pro-inflammatory cytokines in carrageenan-induced paw edema in mice.

Cytokine (pg/mg protein)	IL-1β
Non-Carrageenan	
Control	16.67 ± 1.63
Carrageenan	
Control	29.55 ± 3.06*
Sch A 0.25nmol/kg	16.40 ± 3.09^#^
Sch B 0.25nmol/kg	15.46 ± 1.59^#^
Sch A+Sch B 0.125nmol/kg	18.60 ± 1.90^#^

IL-1β, interleukin 1β.

* Significantly different from the non-carrageenan control;

^#^ significantly different from the carrageenan control.

### Effect of long-term treatment with Sch A or Sch B on Con A-activated splenocytes isolated from treated mice

**Fig 12:**
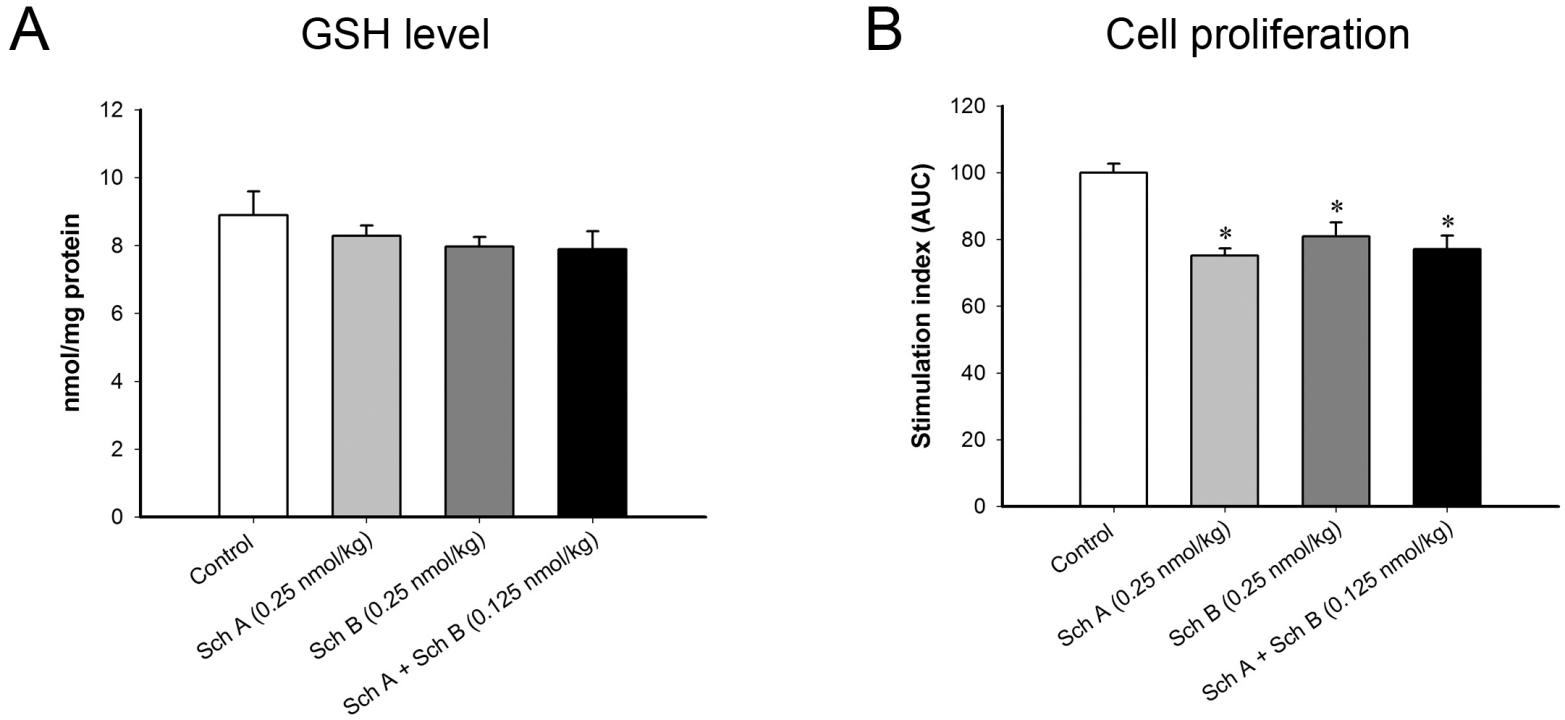
Effects of long-term treatment with Sch A and Sch B in concanavalin A (Con A)-activated splenocytes isolated from Sch A/Sch B-treated mice. Female ICR mice were administered Sch A or Sch B orally [alone (0.25 nmol/kg/d) or in combination (0.125 nmol/kg/d) × 5 d], as described in Materials and Methods. Splenocytes were isolated from the spleen of Sch A/Sch B-treated mice, as described in Materials and Methods. (A) The isolated splenocytes were lysed and the levels of GSH in the cell lysate were measured. (B) Isolated splenocytes were incubated with Con A (1, 2 and 4 μg/mL) for 72 h. The Con A-induced proliferation of splenocytes was measured by MTT assay. The area under the curve (AUC) plotting the absorbance at 570 nm against the concentration of Con A was calculated and expressed as stimulation index. Data are expressed % control by normalizing with the vehicle control group. * Significantly different from the control group.

To confirm the results obtained from the *in vitro* splenocyte study, an *ex vivo* model was adopted to investigate the effect of Sch A/Sch B pretreatment on Con A-stimulated splenocyte proliferation in mice. Long-term treatment with Sch A, Sch B and Sch A+Sch B did not produce any detectable change in cellular GSH levels in splenocytes isolated from pretreated mice. However, long-term treatment with Sch A, Sch B and Sch A+Sch B did inhibit the Con A-activated splenocyte proliferation (25, 19 and 23%, respectively) (Fig 12). The inhibition of Con A-stimulated splenocyte proliferation by Sch A, Sch B or Sch A+Sch B pretreatment was found to be associated with decreases in the release of T helper cell 1 cytokines, such as IL-2 (60, 54 and 63%), TNF-α (42, 20 and 62%) and IFN-γ (89, 118 and 85%) (Table 7).

**Table 7:** Effect of long-term treatment with Sch A and Sch B on T helper cell 1 cytokines in concanavalin A (Con A) -activated splenocytes isolated from Sch-treated mice.

Cytokines of T Helper Cell 1 (Th1) pg/mL	IL-2	TNF-α	INF-γ
Non-Concanavalin A			
Control	1.5 ± 0.6	2.6 ± 0.8	2.2 ± 0.7
Concanavalin A			
Control	144.7 ± 24.5*	8.6 ± 0.4*	5.2 ± 0.3*
Sch A 0.25nmol/kg	58.7 ± 11.5^#^	6.1 ± 0.8^#^	2.5 ± 0.8^#^
Sch B 0.25nmol/kg	67.0 ± 15.1^#^	6.8 ± 1.1^#^	1.7 ± 0.3^#^
Sch A+Sch B 0.125nmol/kg	54.1 ± 16.3^#^	4.9 ± 0.7^#^	2.7 ± 1.0^#^

IL-2, interleukin 2; TNF-α, tumor necrosis factor α; interferon γ, INF-γ.

* Significantly different from the non- control;

^#^ significantly different from the carrageenan control.

## Discussion

The LPS-activated signal transduction in macrophages, which involves a cascade of the Toll-like receptor 4/JNK/P38/NF-κB pathway, is commonly investigated in cell models for assessing anti-inflammatory activities of a variety of candidate compounds [22–24]. Consistent with the traditional use of Schisandra fruits for the treatment of inflammation in Chinese medicine, both Sch A and Sch B were found to reduce the extent of inflammation in LPS-activated RAW264.7 macrophages, with Sch A being more potent. Consistent with this finding, a recent study has shown that Sch A can reduce the release of NO, TNF-α and IL-6 in LPS-activated BV-2 microglia cells as well as primary microglia cells via inhibition of TRAF6/NF-κB/Jak2/Stat3 signaling pathways [25]. In the present study, the anti-inflammatory action produced by Sch A and Sch B was associated with their ability to increase GST activity and decrease cellular GSH level in macrophages, with the degree of stimulation/depletion produced by Sch A being more pronounced. In agreement with the greater anti-inflammatory action produced by Sch A, acute treatment with Sch A (but not Sch B) was found to decrease GSH levels in isolated lymphocytes from the blood of treated mice. In addition, acute treatment with Sch A reduced the extent of carrageenan-induced paw edema in mice. While it has been suggested that GSH depletion and anti-inflammatory activity may be causally linked to the modification of a redox-sensitive regulatory cysteine residue in NF-κB under the conditions of reduced cellular GSH content [26], recent studies have also demonstrated the involvement of GST in the regulation of a pro-inflammatory signal transduction cascade in cultured macrophages. As such, the recombinant protein glutathione S-transferase P1 (GSTP1) can suppress levels of iNOS and COX2 in LPS-activated RAW264.7 macrophages [27]. In addition, the schistosome glutathione S-transferase (P28GST) was found to reduce the extent of intestinal inflammation in experimental colitis [28]. These findings are in good agreement with our observation that the activation of GST induced by Sch A/Sch B is associated with the reduction in the extent of the inflammatory response in cultured macrophages. While the catalytic action of GST may lead to the consumption of cellular GSH and hence its depletion, the inhibition of GST did not completely abrogate the GSH depletion induced by Sch A/Sch B in macrophages. The biochemical mechanism underlying the Sch A/Sch B-induced GSH depletion remains to be elucidated. Recently, reactions leading to S-glutathionylation and de-glutathionylation have been shown to be a novel regulatory mechanism of protein activities under conditions of oxidative stress [29]. Given that GSH/GST is a critical substrate/enzyme combination in S-glutathionylation, it is possible that this process may be involved in the anti-inflammatory action of Sch A/Sch B. The differences in potency of Sch A and Sch B in causing GSH depletion and hence anti-inflammation may be related to the difference their chemical structure. While Sch A possesses 2 methyloxy groups at the C4’ and C5’ positions of the phenyl ring, Sch B contains a methylenedioxy ring moiety. As such, the difference between Sch A and Sch B in the interaction with target protein(s) and/or in the production of reactive metabolites may contribute to the difference in their effectiveness in GSH depletion and anti-inflammation.

Accumulating experimental evidence has demonstrated the involvement of an Nrf2-driven antioxidant response in the suppression of inflammation. Consistent with our previous finding in cultured murine hepatocytes and cardiomyocytes [12,13], the results showed that Sch B (but not Sch A) caused a Nrf2-driven TRX induction in macrophages. Our finding indicated that the anti-inflammatory action of Sch B is paralleled by the induction of TRX in LPS-activated macrophages. While the long-term treatment with Sch B (but not Sch A) induced an antioxidant response, both long-term Sch A and Sch B treatments suppressed paw edema in carrageenan-injected mice. Without the induction of an antioxidant response, Sch A, which is likely accumulated in paw tissue with long-term treatment, may act directly on macrophages/lymphocytes in producing the anti-inflammatory action *in vivo*. Given that the maximum achievable blood concentration of Sch B in mice is around 10 μM (unpublished data) which is lower than the effective concentrations of Sch B (25–50 μM) in *in vitro* assays, the immune cells may interact synergistically in *in vivo* condition in response to Sch B and thereby produce the anti-inflammatory action.

The ability of Sch B to induce an antioxidant response is likely mediated by the CYP-catalyzed metabolism of Sch B, with the production of signaling ROS resulting in activation of Nrf2 [12,13]. This was corroborated by the observation that co-incubation with a CYP inhibitor (or NAC) as well as Nrf2 knockdown could attenuate the anti-inflammatory action elicited by Sch B. A recent finding in our laboratory has shown that the Sch B-induced expression of TRX can inhibit the activation of NLRP3 inflammasome in cultured mouse macrophages [30]. Taken together, the anti-inflammatory action of Sch B may, at least in part, be secondary to the induction of an antioxidant response.

Con A, a lectin isolated from jack-beans, is a T lymphocyte-specific antigen that preferentially triggers the differentiation and proliferation of immature lymphocytes into T lymphocytes [31]. In this regard, incubation with Con A can induce the differentiation and proliferation of splenocytes (a mixed population of immature T and B lymphocytes) into T lymphocytes. Among the T cell sub-populations, T1 helper (Th1) cells and T2 (Th2) helper cells were found to be involved in the regulation of inflammatory response via an interaction with macrophages [32]. While Th1 cells (which mainly secrete IL-2, TNF-α and IFN-γ) are pro-inflammatory, Th2 cells (which primarily secrete IL-4, IL-5, IL-10 and IL-13) are anti-inflammatory [33]. Our results indicate that incubation with Sch A (but not Sch B) can induce GSH depletion in splenocytes as well as the associated inhibition of the release of IL-2 and cell proliferation in Con A-activated splenocytes. The suppression of IL-2 release by Sch A in Con A-activated splenocytes may be causally related to the inhibition of IL-2 release from Th1 cells. This postulation is supported by the results obtained from the *ex vivo* study that levels of Th1 cytokines (such as IL-2, TNFα and IFNγ) were suppressed in Con A-activated splenocytes isolated from Sch A-pretreated mice. Similar to the results obtained from the study in macrophages, the Sch A-induced GSH depletion seems to be associated with immunosuppression in Con A-activated splenocytes. In this regard, it has been reported that the proliferation of cultured murine lymphocytes is dependent on the availability of cellular GSH [34]. In addition, Th1 cells are pro-inflammatory by virtue of cytokine (IL-2, TNF-α and IFN-γ) secretion. Accordingly, anti-inflammatory action of Sch A may be mediated by regulatory T helper cells which can modulate the activity of macrophages.

Given that the differential biochemical mechanism underlying the anti-inflammatory action of Sch A (mainly involving GST activation/ GSH depletion) and Sch B (mainly via the induction of an antioxidant response), whether or not a synergistic effect can be produced by the combined treatment with Sch A and Sch B, is of considerable pharmacological interest. Unexpectedly, under the present experimental conditions, no synergistic anti-inflammatory effects were observed for Sch A and Sch B in either *in vitro* or in *in vivo* assay systems. The possibility of synergism produced by Sch A and Sch B in combination may possibly be revealed in other experimental models of inflammation.

**Fig 13:**
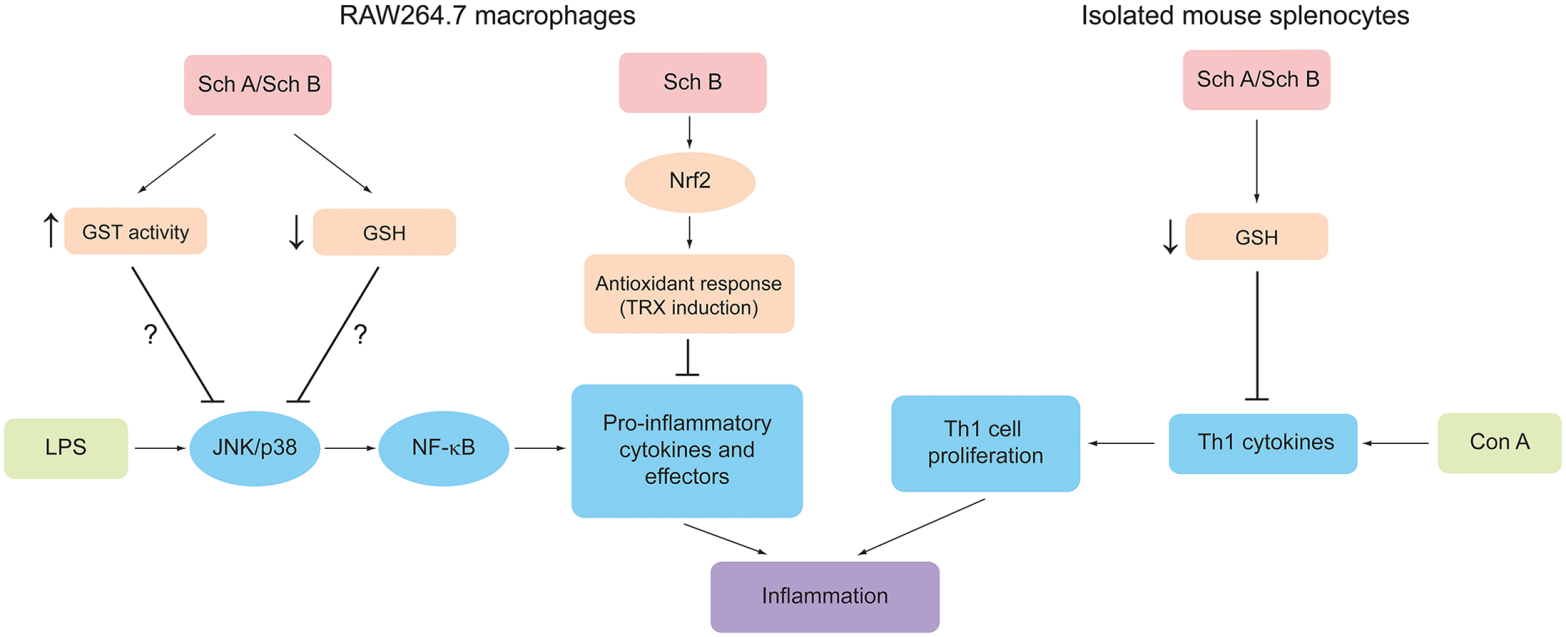
A proposed mechanism underlying the anti-inflammatory actions of Sch A and Sch B. Both Sch A and Sch B can elicit a direct anti-inflammatory action via the activation of GST and depletion of GSH, with the resultant suppression of a pro-inflammatory signaling cascade and pro-inflammatory cytokines as well as effectors in LPS-activated macrophages. Sch B can also induce an indirect anti-inflammatory response via the induction of Nrf2-mediated expression of TRX in LPS-activated macrophages. Sch A and Sch B can suppress the proliferation of Th1 cells (which promotes inflammation) via the depletion of GSH and a reduction in the release of Th1 cytokines in Con A-activated T lymphocytes. *Abbreviations*: Sch A, schisandrin A; Sch B, schisandrin B; GST, glutathione S-transferase; GSH, reduced glutathione; LPS, lipopolysaccharide; JNK, c-Jun N-terminal kinase; p38, p38 kinase; NF-κB, nuclear factor κB; Nrf2, nuclear factor erythroid 2-related factor 2; TRX, thioredoxin; Con A, concanavalin A; Th1, T helper1 subtype.

In conclusion, taken together, our results have demonstrated for the first time that Sch A/Sch B-induced GST activation and glutathione depletion are likely involved in their anti-inflammatory actions *in vitro* and *in vivo* (Fig 13). Further study is needed to elucidate the mechanism of GSH depletion induced by Sch A and Sch B. Given that the ability of Sch A to cause GSH depletion and reduce the extent of inflammation is greater than that of Sch B, a structure-activity relationship study of Sch A and Sch B in S-glutathionylation and de-glutathionylation of inflammatory regulators in relation to their anti-inflammatory actions in macrophages and lymphocytes seems warranted.

## Supporting Information

S1 Fig Effect of quinidine [a glutathione S-transferase (GST) inhibitor] on the anti-inflammatory action of Sch A and Sch B in lipopolysaccharide (LPS)-activated RAW264.7 macrophages. RAW264.7 macrophages were co-incubated with quinidine (18 μM) during the incubation with Sch A or Sch B. After one h co-incubation, GST activity was measured (left; upper panel). After 6 h, the GSH level was also measured (left; lower panel). Data are expressed as % non-quinidine control by normalizing relative to non-quinidine-incubated cells (left panels). Value given are means ± SEM, with n = 3–5. * Significantly different from the non-quinidine control; # significantly different from the quinidine control. After 6 h co-incubation, RAW264.7 macrophages were stimulated with LPS (1 μg/mL) for 18 h and 24 h, for the measurement of iNOS and NO levels, respectively. Levels of iNOS and NO were measured as described in Fig 6. Data are expressed as % non-LPS control by normalizing relative to non-quinidine incubated controls without LPS stimulation. Value given are means ± SEM, with n = 3–5. * Significantly different from the non-quinidine control group; # significantly different from the non-quinidine group with LPS stimulation; + significantly different from the quinidine control group.
(TIF)

S2 Fig Effect of N-acetylcysteine (NAC, a thiol antioxidant) on the anti-inflammatory action produced by Sch A and Sch B in lipopolysaccharide (LPS)-activated RAW264.7 macrophages. RAW264.7 macrophages were co-incubated with NAC (5 mM) during the 6-h incubation with Sch A or Sch B. GSH levels were measured (left panel). Data are expressed as % non-NAC control by normalizing relative to non-NAC incubated control cells. Value given are means ± SEM, with n = 3–5. * Significantly different from the non-NAC control; # significantly different from the NAC control. After the 6-h co-incubation, RAW264.7 macrophages were challenged with LPS (1 μg/mL) for 18 or 24 h, for the measurement of inducible nitric oxide synthase (iNOS) and nitric oxide (NO) levels, respectively. Levels of iNOS and NO were measured as described in Fig 6. Data are expressed as % non-LPS control by normalizing relative to non-NAC incubated controls without LPS stimulation. Value given are means ± SEM, with n = 3–5. * Significantly different from the non-NAC control group; # significantly different from the non-NAC group with LPS stimulation; + significantly different from the NAC control group; ^ significantly different from the NAC group with LPS stimulation.
(TIF)

S3 Fig Effect of Trolox (a non-thiol antioxidant) on the anti-inflammatory actions of Sch A and Sch B in lipopolysaccharide (LPS)-activated RAW264.7 macrophages. RAW264.7 macrophages were co-incubated with Trolox (500 μM) during the 6-h incubation with Sch A or Sch B. GSH levels were measured (left panel). Data are expressed as % non-Trolox control by normalizing relative to non-Trolox incubated control cells. Value given are means ± SEM, with n = 3–5. * Significantly different from the non-Trolox control; # significantly different from the Trolox control. After the 6-h co-incubation, RAW264.7 macrophages were challenged with LPS (1 μg/mL) for 18 or 24 h, for the measurement of inducible nitric oxide synthase (iNOS) and nitric oxide (NO) levels, respectively. Levels of iNOS and NO were measured as described in Fig 6. Data are expressed as % non-LPS control by normalizing relative to non-Trolox incubated controls without LPS stimulation. Value given are means ± SEM, with n = 3–5. * Significantly different from the non-Trolox control group; # significantly different from the non-Trolox group with LPS stimulation; + significantly different from the Trolox control group; ^ significantly different from the Trolox group with LPS stimulation.
(TIF)

S4 Fig Effect of 1-aminobenzotriazole (ABT, a cytochrome P450 inhibitor) on the anti-inflammatory action of Sch A or B in lipopolysaccharide (LPS)-activated macrophages. RAW264.7 macrophages were transfected with Nrf2 luciferase reporter, as described in Materials and Methods. The transfected macrophages were co-incubated with ABT (10 mM) during the incubation with Sch A or Sch B for 6 h. At 16 h post- exposure, the luciferase activities in the cell lysates were measured. Nrf2 reporter activities were expressed as % control, by normalizing relative to non-ABT controls (left; upper panel). At 16 h post-exposure, the level of thioredoxin (TRX) was also measured, as described in Fig 10. The content of TRX was normalized relative to the corresponding β-actin level and expressed as % non-ABT control (left; lower panel). Value given are means ± SEM, with n = 3. * Significantly different from the non-ABT control. After the co-incubation with ABT during Sch A or Sch B exposure, the cells were challenged with LPS (1μg/mL) at 16 h post-exposure. Levels of inducible nitric oxide synthase (iNOS) and nitric oxide (NO) were measured at 18 or 24 h, respectively after the LPS challenge, as described in Fig 5. Data are expressed as % non-LPS control by normalizing relative to non-ABT incubated controls without LPS stimulation. Value given are means ± SEM, with n = 3–5. * Significantly different from the non-ABT control group; # significantly different from the non-ABT group with LPS stimulation; + significantly different from the ABT control group; ^ significantly different from the ABT group with LPS stimulation.
(TIF)

S5 Fig Effect of Nrf2 knockdown on the anti-inflammatory action afforded by Sch B in lipopolysaccharide (LPS)-activated macrophages. RAW264.7 macrophages were co-transfected with Nrf2 luciferase reporter as well as siRNA of Nrf2, as described in Materials and Methods. The transfected macrophages were incubated with Sch B (50 μM) for 6 h. Followingt 16 h of incubation, the luciferase activities in the cell lysates were measured. The Nrf2 reporter activity was expressed as % control, by normalizing relative to the value of the non-siRNA control (left; upper panel). Following 16 h of incubation, the level of thioredoxin (TRX) was measured, as described in Fig 7. The amount of TRX was normalized relative to the β-actin level and expressed as % non-siRNA control (left; lower panel). Value given are means ± SEM, with n = 3. * Significantly different from the non-siRNA control. Nrf2 knockdown macrophages were incubated with Sch A and Sch B for 6 h. The cells were then challenged with LPS (1 μg/mL) at 16 h following exposure. Levels of iNOS and NO were measured at 18 or 24 h, respectively, after the LPS challenge, as described in Fig 5. Data are expressed as % non-LPS control by normalizing relative to the value of non-siRNA knockdown controls without LPS challenge. Value given are means ± SEM, with n = 3–5. * Significantly different from the non-siRNA knockdown control group; # significantly different from the non-siRNA knockdown group with LPS challenge; + significantly different from the siRNA knockdown control group; ^ significantly different from the siRNA knockdown group with LPS challenge.
(TIF)

## References

[1] Soehnlein O, Lindbom L. Phagocyte partnership during the onset and resolution of inflammation. Nat Rev Immunol. 2010; 10: 427–439. 10.1038/nri2779 20498669

[2] Portou MJ, Baker D, Abraham D, Tsui J. The innate immune system, toll-like receptors and dermal wound healing: A review. Vascul Pharmacol. 2015; 71: 31–36. 10.1016/j.vph.2015.02.007 25869514

[3] Shi C, Pamer EG. Monocyte recruitment during infection and inflammation. Nat Rev Immunol. 2011; 11: 762–774. 10.1038/nri3070 21984070 PMC3947780

[4] Loddo I, Romano C. Inflammatory Bowel Disease: Genetics, Epigenetics, and Pathogenesis. Front Immunol. 2015; 6: 551 10.3389/fimmu.2015.00551 eCollection 2015. 26579126 PMC4629465

[5] Koenitzer JR, Freeman BA. Redox signaling in inflammation: interactions of endogenous electrophiles and mitochondria in cardiovascular disease. Ann N Y Acad Sci. 2010; 1203: 45–52. 10.1111/j.1749-6632.2010.05559.x 20716282 PMC4106461

[6] Reuter S, Gupta SC, Chaturvedi MM, Aggarwal BB. Oxidative stress, inflammation, and cancer: how are they linked? Free Radic Biol Med. 2010; 49: 1603–1616. 10.1016/j.freeradbiomed.2010.09.006 20840865 PMC2990475

[7] Balmus IM, Ciobica A, Trifan A, Stanciu C. The implications of oxidative stress and antioxidant therapies in Inflammatory Bowel Disease: Clinical aspects and animal models. Saudi J Gastroenterol. 2016; 22: 3–17. 10.4103/1319-3767.173753 26831601 PMC4763525

[8] Straub RH, Schradin C. Chronic inflammatory systemic diseases—an evolutionary trade-off between acutely beneficial but chronically harmful programs. Evol Med Public Health. 2016; In press. 10.1093/emph/eow001 PMC4753361 26817483

[9] Kauppinen A, Paterno JJ, Blasiak J, Salminen A, Kaarniranta K. Inflammation and its role in age-related macular degeneration. Cell Mol Life Sci. 2016; In press. 10.1007/s00018-016-2147-8 PMC4819943 26852158

[10] Salminen A, Huuskonen J, Ojala J, Kauppinen A, Kaarniranta K, Suuronen T. Activation of innate immunity system during aging: NF-kB signaling is the molecular culprit of inflamm-aging. Ageing Res Rev. 2008; 7: 83–105. 17964225 10.1016/j.arr.2007.09.002

[11] Fulop T, Le Page A, Fortin C, Witkowski JM, Dupuis G, Larbi A. Cellular signaling in the aging immune system. Curr Opin Immunol. 2014; 29: 105–111. 10.1016/j.coi.2014.05.007 24934647

[12] Leong PK, Chiu PY, Chen N, Leung H, Ko KM. Schisandrin B elicits a glutathione antioxidant response and protects against apoptosis via the redox-sensitive ERK/Nrf2 pathway in AML12 hepatocytes. Free Radic Res. 2011; 45: 483–495. 10.3109/10715762.2010.550917 21250784

[13] Chiu PY, Chen N, Leong PK, Leung HY, Ko KM. Schisandrin B elicits a glutathione antioxidant response and protects against apoptosis via the redox-sensitive ERK/Nrf2 pathway in H9c2 cells. Mol Cell Biochem. 2011; 350: 237–250. 10.1007/s11010-010-0703-3 21193948

[14] Lu SC. Glutathione synthesis. Biochim Biophys Acta. 2013; 1830: 3143–3153. 10.1016/j.bbagen.2012.09.008 22995213 PMC3549305

[15] Lu J, Holmgren A. The thioredoxin antioxidant system. Free Radic Biol Med. 2014; 66: 75–87. 10.1016/j.freeradbiomed.2013.07.036 23899494

[16] Huyke C, Engel K, Simon-Haarhaus B, Quirin KW, Schempp CM. Composition and biological activity of different extracts from Schisandra sphenanthera and Schisandra chinensis. Planta Med. 2007; 73: 1116–1126. 17611932 10.1055/s-2007-981559

[17] Oh SY, Kim YH, Bae DS, Um BH, Pan CH, Kim CY, et al Anti-inflammatory effects of gomisin N, gomisin J, and schisandrin C isolated from the fruit of Schisandra chinensis. Biosci Biotechnol Biochem. 2010; 74: 285–291. 20139628 10.1271/bbb.90597

[18] Checker R, Patwardhan RS, Sharma D, Menon J, Thoh M, Bhilwade HN, et al Schisandrin B exhibits anti-inflammatory activity through modulation of the redox-sensitive transcription factors Nrf2 and NF-κB. Free Radic Biol Med. 2012; 53: 1421–1430. 10.1016/j.freeradbiomed.2012.08.006 22917978

[19] Chen N, Ko M. Schisandrin B-induced glutathione antioxidant response and cardioprotection are mediated by reactive oxidant species production in rat hearts. Biol Pharm Bull. 2010; 33: 825–829. 20460761 10.1248/bpb.33.825

[20] Chiu PY, Tang MH, Mak DH, Poon MK, Ko KM. Hepatoprotective mechanism of schisandrin B: role of mitochondrial glutathione antioxidant status and heat shock proteins. Free Radic Biol Med. 2003; 35: 368–380. 12899939 10.1016/s0891-5849(03)00274-0

[21] Griffith OW. Determination of glutathione and glutathione disulfide using glutathione reductase and 2-vinylpyridine. Anal Biochem. 1980; 106: 207–212. 7416462 10.1016/0003-2697(80)90139-6

[22] Murakami Y, Ishii H, Takada N, Tanaka S, Machino M, Ito S, et al Comparative anti-inflammatory activities of curcumin and tetrahydrocurcumin based on the phenolic O-H bond dissociation enthalpy, ionization potential and quantum chemical descriptor. Anticancer Res. 2008; 28: 699–707. 18507010

[23] Han AR, Kang YJ, Windono T, Lee SK, Seo EK. Prenylated flavonoids from the heartwood of Artocarpus communis with inhibitory activity on lipopolysaccharide-induced nitric oxide production. J Nat Prod. 2006; 69: 719–721. 16643064 10.1021/np0600346

[24] Fu JJ, Qin JJ, Zeng Q, Huang Y, Jin HZ, Zhang WD. Four new sesquiterpenoids from the roots of Incarvillea arguta and their inhibitory activities against lipopolysaccharide-induced nitric oxide production. Chem Pharm Bull (Tokyo). 2010; 58: 1263–1266. 20823614 10.1248/cpb.58.1263

[25] Song F, Zeng K, Liao L, Yu Q, Tu P, Wang X. Schizandrin A Inhibits Microglia-Mediated Neuroninflammation through Inhibiting TRAF6-NF-κB and Jak2-Stat3 Signaling Pathways. PLoS One. 2016; 11: e0149991 10.1371/journal.pone.0149991 eCollection 2016. 26919063 PMC4768966

[26] Song M, Kellum JA, Kaldas H, Fink MP. Evidence that glutathione depletion is a mechanism responsible for the anti-inflammatory effects of ethyl pyruvate in cultured lipopolysaccharide-stimulated RAW 264.7 cells. J Pharmacol Exp Ther. 2004; 308: 307–316. 14569076 10.1124/jpet.103.056622

[27] Luo L, Wang Y, Feng Q, Zhang H, Xue B, Shen J, et al Recombinant protein glutathione S-transferases P1 attenuates inflammation in mice. Mol Immunol. 2009; 46: 848–857. 10.1016/j.molimm.2008.09.010 18962899

[28] Driss V, El Nady M, Delbeke M, Rousseaux C, Dubuquoy C, Sarazin A, et al The schistosome glutathione S-transferase P28GST, a unique helminth protein, prevents intestinal inflammation in experimental colitis through a Th2-type response with mucosal eosinophils. Mucosal Immunol. 2015; In press. 10.1038/mi.2015.62 PMC4801903 26174763

[29] Dalle-Donne I, Rossi R, Giustarini D, Colombo R, Milzani A. S-glutathionylation in protein redox regulation. Free Radic Biol Med. 2007; 43: 883–898. 17697933 10.1016/j.freeradbiomed.2007.06.014

[30] Leong PK, Ko KM. Schisandrin B induces an Nrf2-mediated thioredoxin expression and suppresses the activation of inflammasome in vitro and in vivo. Biofactors. 2015; 41: 314–323. 10.1002/biof.1224 26307448

[31] Palacios R. Concanavalin A triggers T lymphocytes by directly interacting with their receptors for activation. J Immunol. 1982; 128: 337–342. 6459373

[32] Mosser DM, Edwards JP. Exploring the full spectrum of macrophage activation. Nat Rev Immunol. 2008; 8: 958–969. 10.1038/nri2448 19029990 PMC2724991

[33] Berger A. Th1 and Th2 responses: what are they? BMJ. 2000; 321: 424 10938051 10.1136/bmj.321.7258.424 PMC27457

[34] Hamilos DL, Zelarney P, Mascali JJ. Lymphocyte proliferation in glutathione-depleted lymphocytes: direct relationship between glutathione availability and the proliferative response. Immunopharmacology. 1989; 18: 223–235. 2575086 10.1016/0162-3109(89)90020-9

