# Supplementary figures and images for: Differential Action between Schisandrin A and Schisandrin B in Eliciting an Anti-Inflammatory Action: The Depletion of Reduced Glutathione and the Induction of an Antioxidant Response
Source: PLoS One. 2016 May 19;11(5):e0155879. doi: 10.1371/journal.pone.0155879 (PMC4873034; doi:10.1371/journal.pone.0155879)

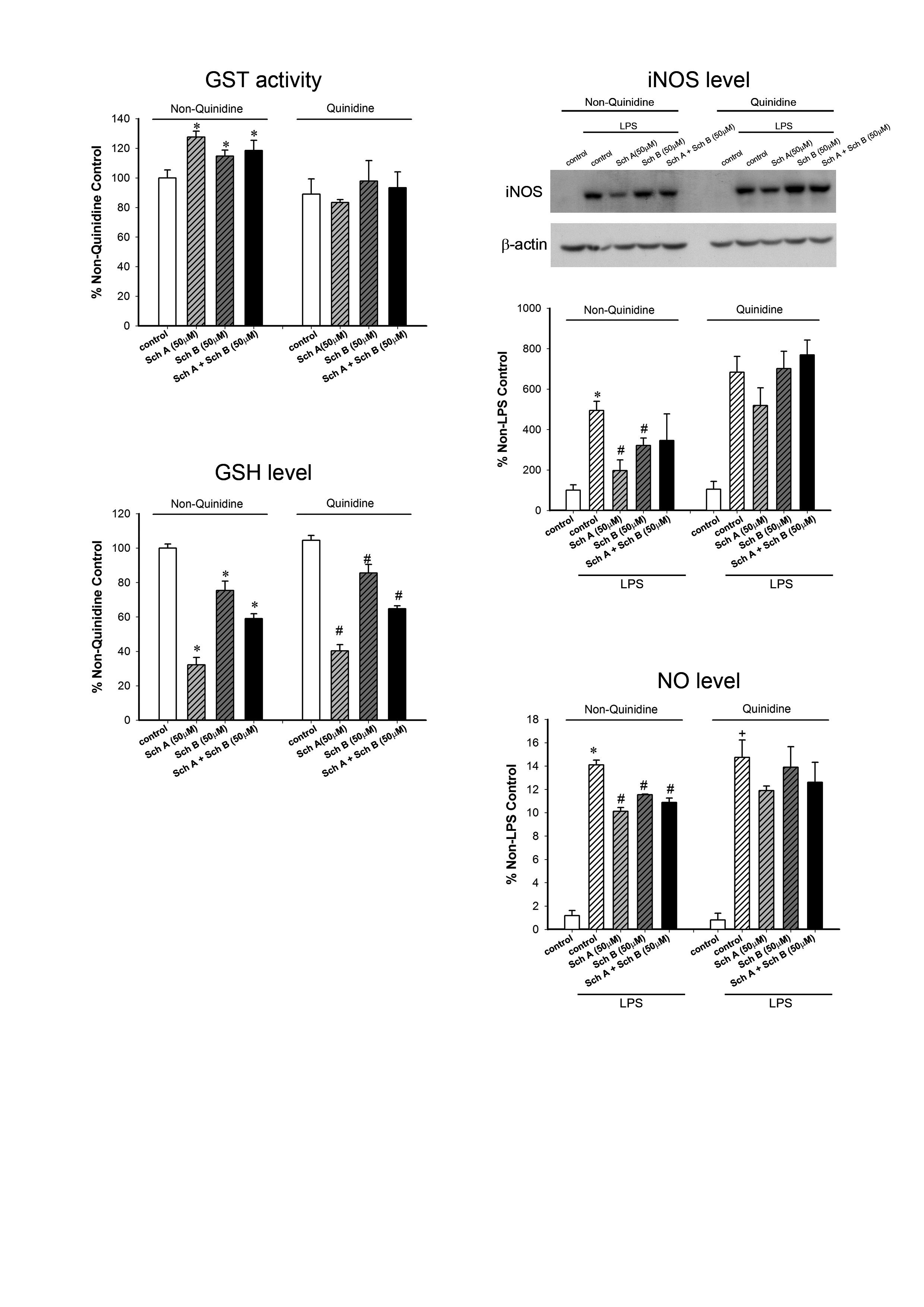

Supplement: S1 Fig — RAW264.7 macrophages were co-incubated with quinidine (18 μM) during the incubation with Sch A or Sch B. After one h co-incubation, GST activity was measured (left; upper panel). After 6 h, the GSH level was also measured (left; lower panel). Data are expressed as % non-quinidine control by normalizing relative to non-quinidine-incubated cells (left panels). Value given are means ± SEM, with n = 3–5. * Significantly different from the non-quinidine control; # significantly different from the quinidine control. After 6 h co-incubation, RAW264.7 macrophages were stimulated with LPS (1 μg/mL) for 18 h and 24 h, for the measurement of iNOS and NO levels, respectively. Levels of iNOS and NO were measured as described in Fig 6. Data are expressed as % non-LPS control by normalizing relative to non-quinidine incubated controls without LPS stimulation. Value given are means ± SEM, with n = 3–5. * Significantly different from the non-quinidine control group; # significantly different from the non-quinidine group with LPS stimulation; + significantly different from the quinidine control group. (TIF) [file pone.0155879.s001.tif]

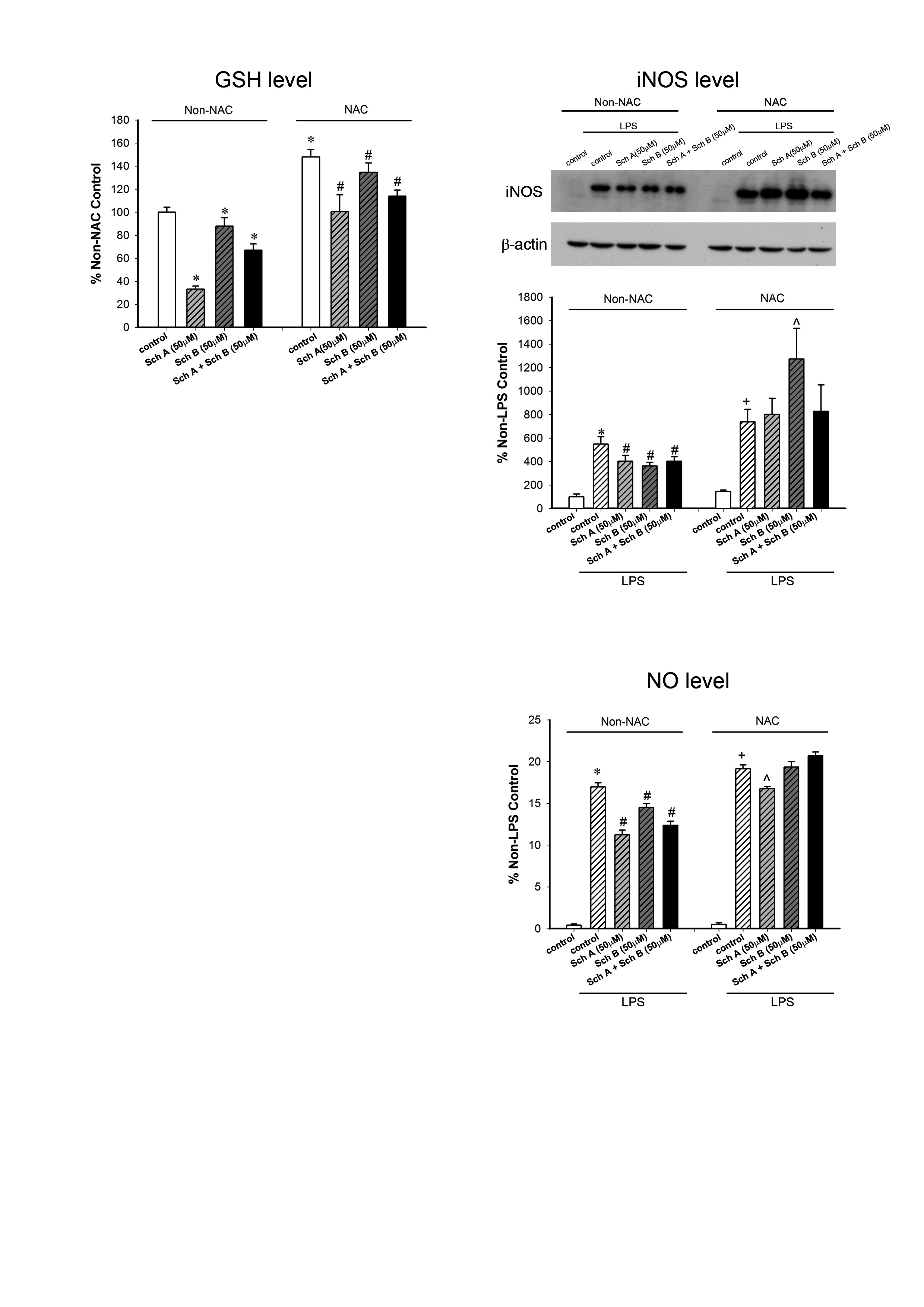

Supplement: S2 Fig — RAW264.7 macrophages were co-incubated with NAC (5 mM) during the 6-h incubation with Sch A or Sch B. GSH levels were measured (left panel). Data are expressed as % non-NAC control by normalizing relative to non-NAC incubated control cells. Value given are means ± SEM, with n = 3–5. * Significantly different from the non-NAC control; # significantly different from the NAC control. After the 6-h co-incubation, RAW264.7 macrophages were challenged with LPS (1 μg/mL) for 18 or 24 h, for the measurement of inducible nitric oxide synthase (iNOS) and nitric oxide (NO) levels, respectively. Levels of iNOS and NO were measured as described in Fig 6. Data are expressed as % non-LPS control by normalizing relative to non-NAC incubated controls without LPS stimulation. Value given are means ± SEM, with n = 3–5. * Significantly different from the non-NAC control group; # significantly different from the non-NAC group with LPS stimulation; + significantly different from the NAC control group; ^ significantly different from the NAC group with LPS stimulation. (TIF) [file pone.0155879.s002.tif]

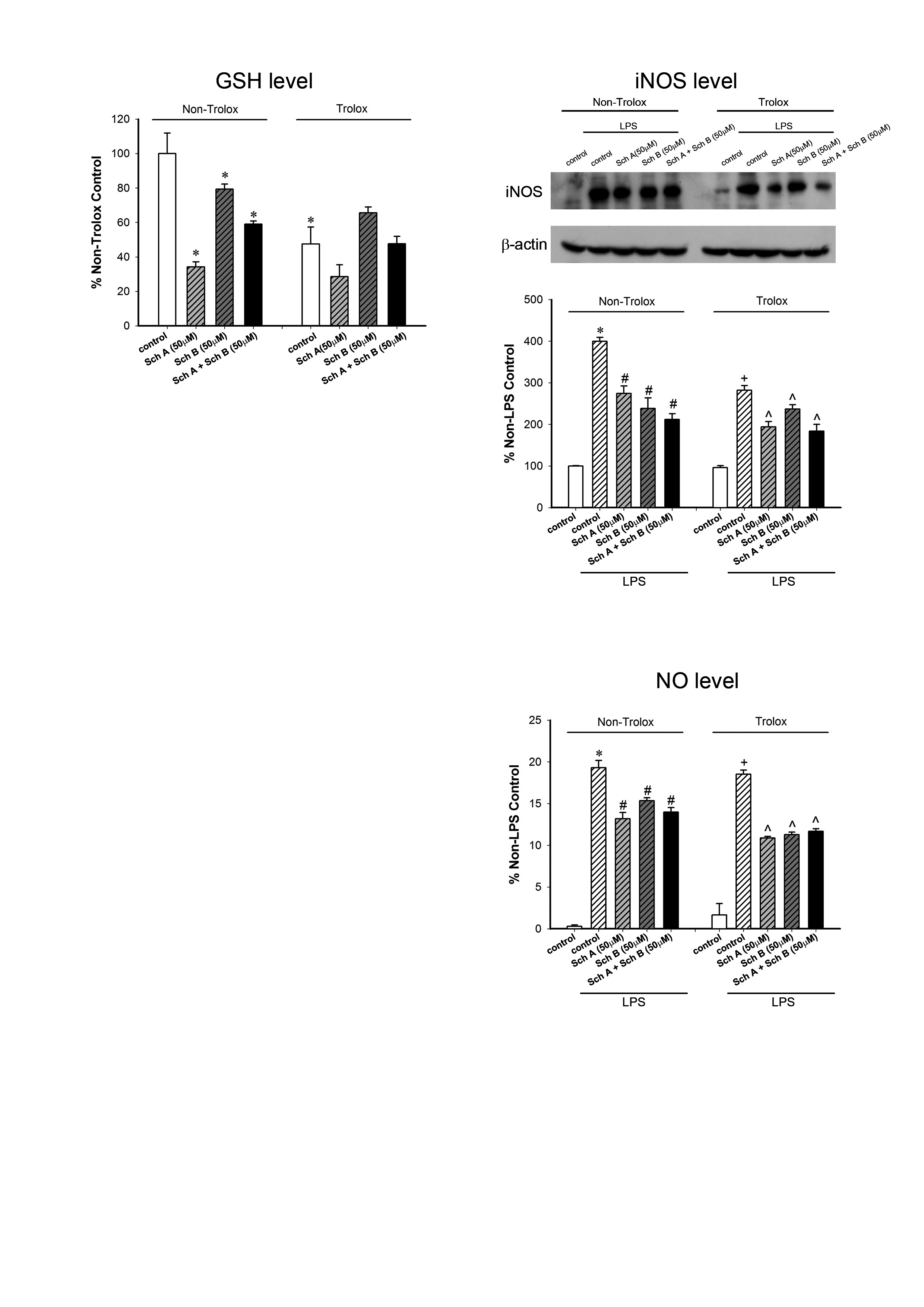

Supplement: S3 Fig — RAW264.7 macrophages were co-incubated with Trolox (500 μM) during the 6-h incubation with Sch A or Sch B. GSH levels were measured (left panel). Data are expressed as % non-Trolox control by normalizing relative to non-Trolox incubated control cells. Value given are means ± SEM, with n = 3–5. * Significantly different from the non-Trolox control; # significantly different from the Trolox control. After the 6-h co-incubation, RAW264.7 macrophages were challenged with LPS (1 μg/mL) for 18 or 24 h, for the measurement of inducible nitric oxide synthase (iNOS) and nitric oxide (NO) levels, respectively. Levels of iNOS and NO were measured as described in Fig 6. Data are expressed as % non-LPS control by normalizing relative to non-Trolox incubated controls without LPS stimulation. Value given are means ± SEM, with n = 3–5. * Significantly different from the non-Trolox control group; # significantly different from the non-Trolox group with LPS stimulation; + significantly different from the Trolox control group; ^ significantly different from the Trolox group with LPS stimulation. (TIF) [file pone.0155879.s003.tif]

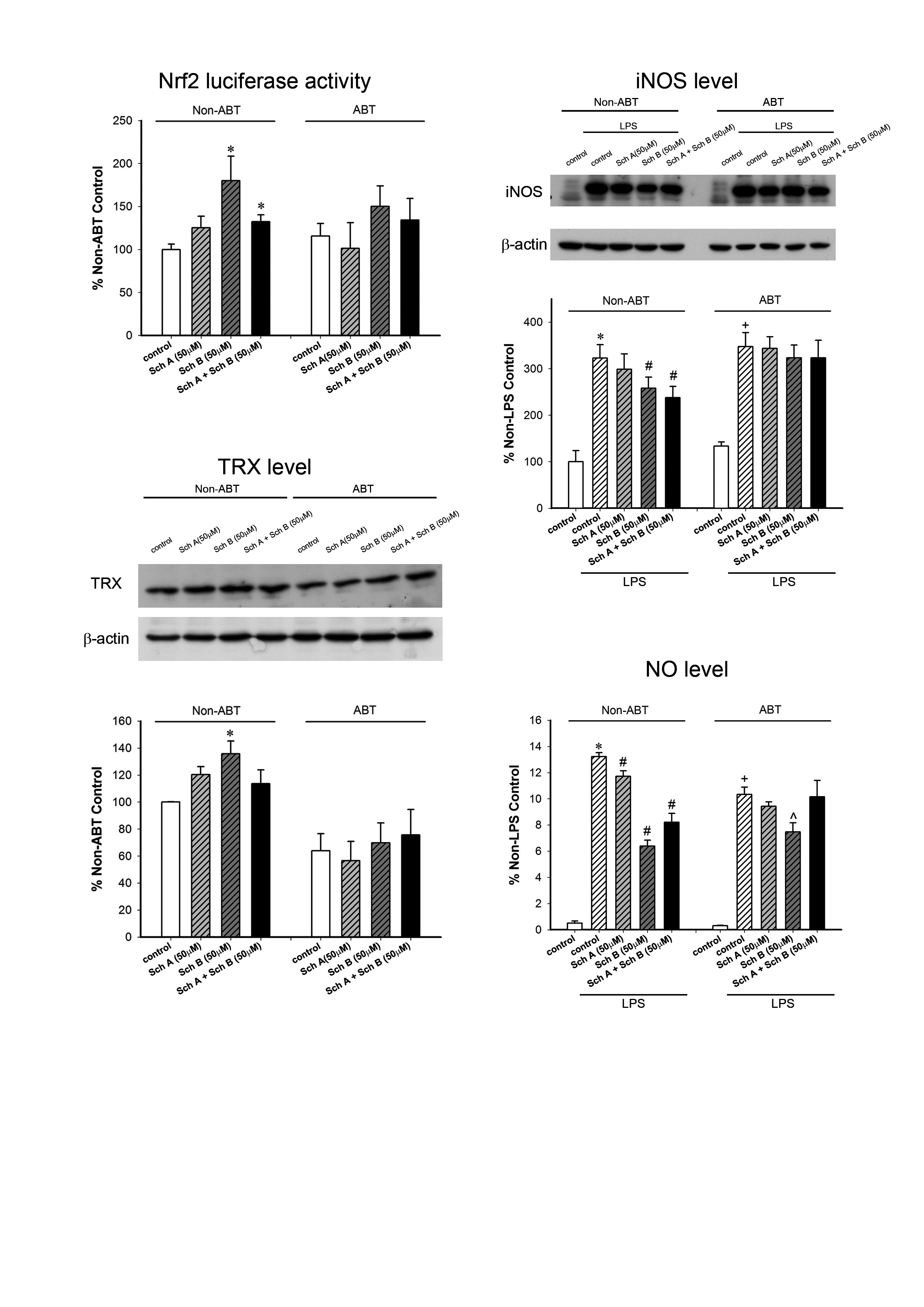

Supplement: S4 Fig — RAW264.7 macrophages were transfected with Nrf2 luciferase reporter, as described in Materials and Methods. The transfected macrophages were co-incubated with ABT (10 mM) during the incubation with Sch A or Sch B for 6 h. At 16 h post- exposure, the luciferase activities in the cell lysates were measured. Nrf2 reporter activities were expressed as % control, by normalizing relative to non-ABT controls (left; upper panel). At 16 h post-exposure, the level of thioredoxin (TRX) was also measured, as described in Fig 10. The content of TRX was normalized relative to the corresponding β-actin level and expressed as % non-ABT control (left; lower panel). Value given are means ± SEM, with n = 3. * Significantly different from the non-ABT control. After the co-incubation with ABT during Sch A or Sch B exposure, the cells were challenged with LPS (1μg/mL) at 16 h post-exposure. Levels of inducible nitric oxide synthase (iNOS) and nitric oxide (NO) were measured at 18 or 24 h, respectively after the LPS challenge, as described in Fig 5. Data are expressed as % non-LPS control by normalizing relative to non-ABT incubated controls without LPS stimulation. Value given are means ± SEM, with n = 3–5. * Significantly different from the non-ABT control group; # significantly different from the non-ABT group with LPS stimulation; + significantly different from the ABT control group; ^ significantly different from the ABT group with LPS stimulation. (TIF) [file pone.0155879.s004.tif]

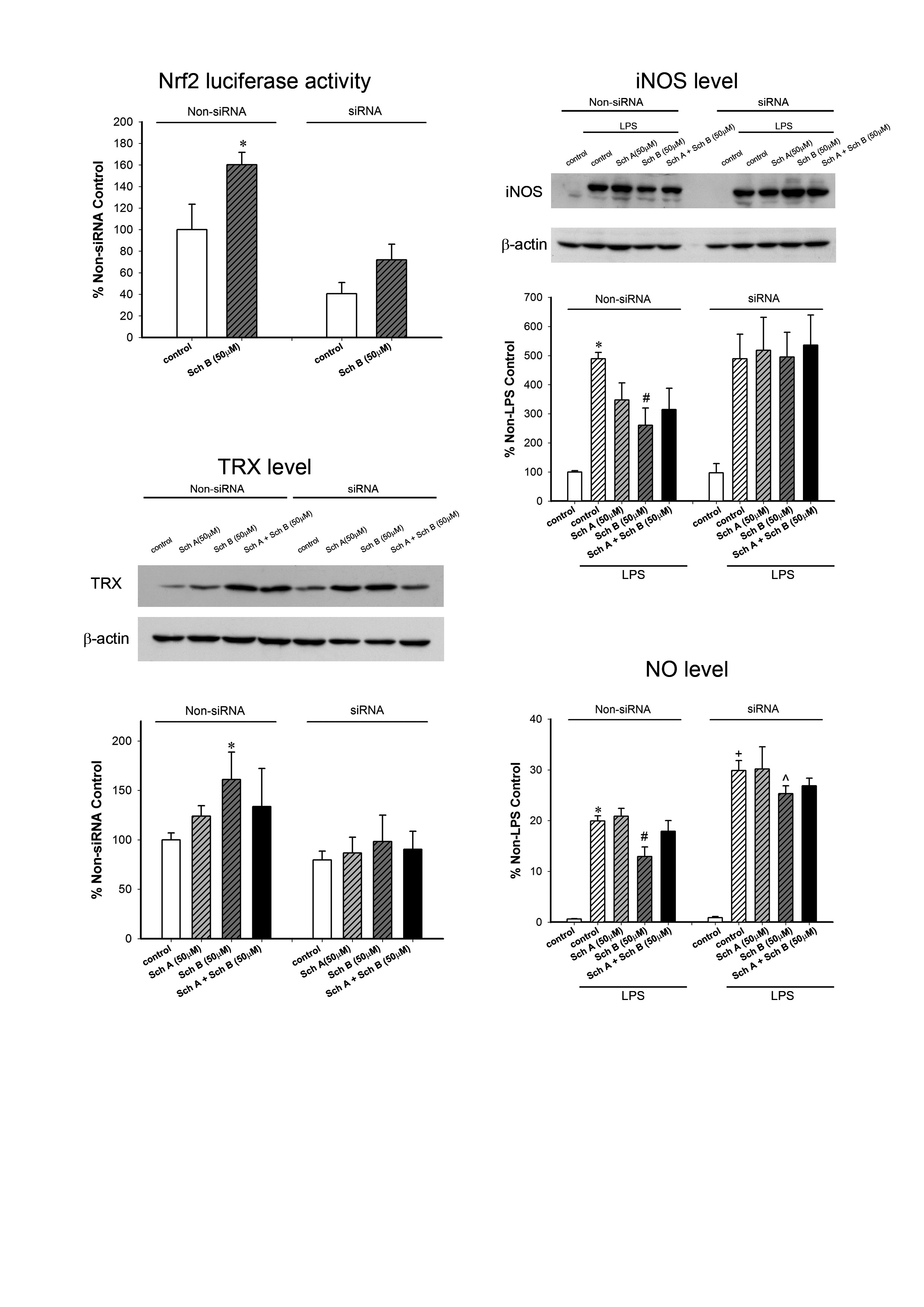

Supplement: S5 Fig — RAW264.7 macrophages were co-transfected with Nrf2 luciferase reporter as well as siRNA of Nrf2, as described in Materials and Methods. The transfected macrophages were incubated with Sch B (50 μM) for 6 h. Followingt 16 h of incubation, the luciferase activities in the cell lysates were measured. The Nrf2 reporter activity was expressed as % control, by normalizing relative to the value of the non-siRNA control (left; upper panel). Following 16 h of incubation, the level of thioredoxin (TRX) was measured, as described in Fig 7. The amount of TRX was normalized relative to the β-actin level and expressed as % non-siRNA control (left; lower panel). Value given are means ± SEM, with n = 3. * Significantly different from the non-siRNA control. Nrf2 knockdown macrophages were incubated with Sch A and Sch B for 6 h. The cells were then challenged with LPS (1 μg/mL) at 16 h following exposure. Levels of iNOS and NO were measured at 18 or 24 h, respectively, after the LPS challenge, as described in Fig 5. Data are expressed as % non-LPS control by normalizing relative to the value of non-siRNA knockdown controls without LPS challenge. Value given are means ± SEM, with n = 3–5. * Significantly different from the non-siRNA knockdown control group; # significantly different from the non-siRNA knockdown group with LPS challenge; + significantly different from the siRNA knockdown control group; ^ significantly different from the siRNA knockdown group with LPS challenge. (TIF) [file pone.0155879.s005.tif]
